# CPT1B K321 crotonylation contributes to cardiac dysfunction in endotoxic shock

**DOI:** 10.1038/s12276-026-01730-2

**Published:** 2026-05-28

**Authors:** Ni Yang, Jingjing Yang, Yang-Fan Xu, Xin-Mei Huang, Ying Gu, Ri Wen, Yu-Hang Yang, Peng-Hui Hao, Xin-Ru Yang, Chun-Feng Liu, Wanshan Ning, Tie-Ning Zhang

**Affiliations:** 1 Department of Pediatrics, PICU, Shengjing Hospital of China Medical University, Shenyang, China; 2 Department of Pulmonary and Critical Care Medicine, the First Affiliated Hospital of Xiamen University, School of Medicine, Xiamen University, Xiamen, China; 3 Department of Endocrinology, Shanghai Fifth People’s Hospital, Fudan University, Shanghai, China; 4 Institute for Clinical Medical Research, the First Affiliated Hospital of Xiamen University, School of Medicine, Xiamen University, Xiamen, China

**Keywords:** Cardiomyopathies, Post-translational modifications

## Abstract

Lysine crotonylation (Kcr) is a novel posttranslational modification that has been proven to have evolutionary conservation. However, the role of protein Kcr in the pathogenesis of endotoxic shock-induced secondary cardiomyopathy is elusive. Here a classic rat model of endotoxic shock was induced by intraperitoneal lipopolysaccharide (LPS) injection. Crotonylproteomic analysis was subsequently performed on myocardial tissues to profile the Kcr modifications that occurred during endotoxic shock, and we found that the Kcr of carnitine palmitoyltransferase 1B (CPT1B) at the K321 site was significantly upregulated in LPS-treated rats. These findings were also confirmed in rat primary cardiomyocytes and H9C2 cells exposed to LPS. Furthermore, we demonstrated that the K321cr of CPT1B impaired CPT1B activity. Moreover, we found that the mutation of K321 to R prevented the mutant CPT1B protein from being crotonylated by LPS, thus alleviating LPS-induced lipid droplet deposition and mitochondrial dysfunction, as reflected by the recovery of ATP generation and mitochondrial membrane potential. Consistently, in vivo cardiac-specific overexpression of CPT1B via an AAV9 vector with a cardiomyocyte-specific promoter (cTnT) also confirmed that the K321-R mutation of CPT1B protected against endotoxic shock-induced secondary cardiomyopathy. Further liquid chromatography–tandem mass spectrometry analysis revealed that the crotonyl-transferases P300 and CBP might be involved in the Kcr of CPT1B. Mechanistically, LPS led to the dissociation of CBP from CPT1B, which promoted the binding of P300 to CPT1B, thereby increasing the level of CPT1B K321cr in H9C2 cells. Taken together, the results of our study revealed the regulatory axis of CPT1B K321 cr/CBP/P300 in LPS-induced endotoxic shock.

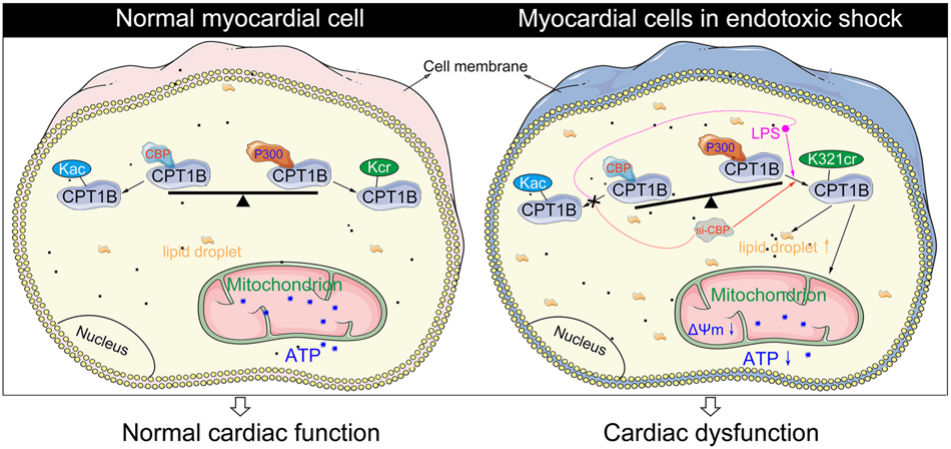

## Crotonylation of CPT1B drives cardiac dysfunction in shock

Endotoxic shock, a severe form of septic shock, is marked by systemic hypotension and reduced heart function due to Gram-negative bacterial toxins. Despite various treatments, effective therapies remain elusive. This study explores the role of lysine crotonylation (Kcr), a protein modification, in endotoxic shock-induced heart dysfunction. Researchers used crotonyl-proteomics to analyze heart tissues from rats subjected to lipopolysaccharide-induced endotoxic shock, identifying increased Kcr at the K321 site of carnitine palmitoyltransferase 1B (CPT1B), a key enzyme in fatty acid metabolism. This modification impaired CPT1B activity, leading to reduced ATP production and heart dysfunction. Introducing a K321R mutation restored enzyme activity and improved heart function in lipopolysaccharide-treated rats. These findings suggest that targeting CPT1B Kcr could offer new therapeutic avenues for treating endotoxic shock-induced heart issues, with future research needed to explore this potential further.

This summary was initially drafted using artificial intelligence, then revised and fact-checked by the author.

## Introduction

Septic shock is a state of acute circulatory failure with a high burden of organ dysfunction and profound cellular metabolic abnormalities during severe infection^1,2^. As a specific etiological subset of septic shock, endotoxic shock is caused primarily by Gram-negative bacterial lipopolysaccharide (LPS) and is characterized by systemic hypotension and reduced myocardial contractility^3,4^. Despite promising preclinical findings, clinical interventions for endotoxic shock, including antibiotics, intravenous immunoglobulin and detoxifying agents such as alkaline phosphatase, have shown limited efficacy^5–7^. Therefore, elucidating the signaling mechanisms that drive endotoxic shock progression is critical for developing effective therapies against this condition and septic shock.

Lysine crotonylation (Kcr) was initially described in 2011 as a protein posttranslational modification (PTM) on the lysine residues of histones^8^. More recent studies have identified Kcr among a large number of nonhistone proteins^9,10^, which are essential for multiple cellular processes, such as remodeling, RNA splicing and metabolism^11^. On the one hand, the Kcr process can be regulated by the cellular concentration of crotonyl-CoA^12^. On the other hand, similar to lysine acetylation (Kac), Kcr is catalyzed mainly by three types of protein kinase^13,14^: writers, which are acyltransferases that are responsible for installing crotonyl groups from modified histones or nonhistones, such as acetyltransferase P300, CREB-binding protein (CBP) and general control nonderepressible 5 (GCN5); erasers, which are deacylases that catalyze the removal of acyl groups from amino acid residues, such as NAD^+^-dependent enzymes class III HDACs (Sirt1-3); and readers, which are the specific proteins responsible for reading the acylation marks, with unknown mechanisms.

Recently, an increasing number of studies have attempted to explore the relationships between Kcr and multiple diseases, including infectious diseases^15^, metabolic diseases^16^, neurological diseases^17^, cancer, and embryonic and reproductive diseases^14^ in animal and cell models. Notably, Kcr has also been shown to be involved in the significant physiological processes of cardiovascular diseases^18,19^. Chen et al.^20^ reported that the Kcr of SERCA2a at Lys120 was significantly increased after cardiac-specific knockout of Sirt1 (Eraser) in mice, which induced cardiac hypertrophy and abnormal energy metabolism in the heart, ultimately leading to arrhythmias. Short-chain enoyl-CoA hydratase (encoded by ECHS1), a hydratase that hydrolyzes crotonyl-CoA, mediates histone crotonylation^21,22^. Mutation of the ECHS1 gene leads to hypertrophic cardiomyopathy^21^. The overexpression of ECHS1 significantly inhibited H3K18cr and H2BK12cr, thereby suppressing Ang II-induced cardiac hypertrophy^18^. In a mouse model of ischemia‒reperfusion, increased general Kcr via sodium crotonate provision or upregulation of the site-specific Kcr of selected mitochondrial protein IDH3a and the cytoskeletal protein TPM1 could protect cardiomyocytes from apoptosis and fibrosis^19^. However, the role of protein Kcr in endotoxic shock-induced secondary cardiomyopathy is poorly understood.

In this study, to identify new therapeutic targets or strategies for prognostic interventions in the context of endotoxic shock, crotonyl-proteomics analysis was performed on myocardial tissues to profile the Kcr modifications that occurred following LPS-induced endotoxic shock. Accordingly, we identified upregulated crotonylation of carnitine palmitoyltransferase 1B (CPT1B) and provided evidence that crotonylation at the K321 site of CPT1B might be crucial to cardiac dysfunction in endotoxic shock.

## Materials and methods

### Cardiomyocyte-specific AAV9 vectors

Cardiomyocyte-specific adeno-associated virus serotype 9 (AAV9) vectors were designed, constructed, and packaged by General Biol (Anhui) Co., Ltd. (China). The vectors utilized the cardiac troponin T (cTnT) promoter to ensure heart-restricted transgene expression. All the constructs were cloned using the SacI restriction site and packaged into AAV9 capsids for enhanced myocardial tropism. Three distinct vectors were produced in this study: AAV9-vector as a negative control; AAV9-Flag-CPT1B K321 for overexpression of wild-type Flag-tagged rat CPT1B; and AAV9-Flag-CPT1B K321R for overexpression of the Flag-tagged K321R mutant.

### Animal experimental protocol

Pathogen-free male Wistar rats (6 weeks old, weighing 170–190 g) were purchased from Liaoning Changsheng Bio Company. The rats were acclimatized for 1 week under standard laboratory conditions with a 12-h light/dark cycle at 22 ± 1 °C. The study protocol was approved by the Ethics Committee of Shengjing Hospital, China Medical University (approval no. 2024PS1503K), and all procedures were carried out in compliance with institutional guidelines and the ARRIVE reporting guidelines 2.0^23^.

#### Rat model of endotoxic shock

Endotoxic shock was established by a single intraperitoneal injection of 20 mg/kg LPS, as previously described^24^. Moreover, saline (2 ml/kg) was administered intraperitoneally to the rats in the control group. Twelve hours after the intraperitoneal injection, the mean arterial blood pressure (MAP) was monitored via femoral artery catheterization. Animals exhibiting a sustained reduction in MAP of approximately 25–30% from baseline levels met the inclusion criteria for endotoxic shock and were included in the study^25^. Following the completion of the hemodynamic assessment, the rats were euthanized. The left ventricle was then rapidly collected for subsequent crotonylproteomics and pathological analyses. The 12-h mortality rate in the LPS group was 15.4% (4/26).

#### Experimental groups and administration

After acclimatization, rats were randomized into the designated experimental groups in accordance with the study protocol outlined below. (1) To delineate the Kcr profile in myocardial tissue during endotoxic shock, a proteomics analysis was performed in which tissues from rats in the LPS group and the control group were compared (*n* = 4 per group). (2) To evaluate myocardial function and CPT1B protein expression in the endotoxic shock model, a subset of rats (*n* = 18 per group) was selected from two established groups: the control group and the LPS group. (3) To evaluate the impact of the CPT1B K321R mutation on baseline cardiac function, a subset of rats (*n* = 18 per group) was selected from three established groups: the AAV9-vector control group, the AAV9-Flag-CPT1B K321 group, and the AAV9-Flag-CPT1B K321R group. The rats in the latter two groups received a tail vein injection of the respective vector (1 × 10¹² genome copies per rat)^26^. Cardiac function was assessed by echocardiography at 2 weeks post injection, after which left ventricular tissue was collected for further analysis. (4) To evaluate the effect of the CPT1B K321R mutation on cardiac function in the endotoxic shock model, the rats were subjected to LPS-induced endotoxic shock two weeks after AAV9 administration. Cardiac function was evaluated by echocardiography 12 h post-LPS injection, and left ventricular tissue was collected for further study. Accordingly, a subset of rats (*n* = 18 per group) was selected from three established groups: the LPS + AAV9-vector control group, the LPS + AAV9-Flag-CPT1B K321 group, and the LPS + AAV9-Flag-CPT1B K321R group. All subsequent echocardiographic and hemodynamic parameter measurements were performed and analyzed by researchers blinded to the treatment groups.

### Echocardiography and hemodynamic parameter measurements

Twelve hours after the intraperitoneal injection of LPS, cardiac function was assessed by echocardiography using a VINNO 6 LAB ultrasound system (Vinno Technology) equipped with an X6-16L linear array transducer. The key parameters measured included heart rate (HR), left ventricular internal diameter in diastole (LVIDd) and systole (LVIDs), ejection fraction (EF) and fractional shortening (FS). After baseline MAP monitoring was performed via femoral artery catheterization, the left ventricle was catheterized to record key hemodynamic parameters using a Bio-signal acquisition and processing system (MD3000-C; Anhui Zhenghua Biological Instrument & Equipment). These included left ventricular end-diastolic pressure (LVEDP), left ventricular end-systolic pressure (LVESP), maximal pressure variation during contraction (+d*P*/d*t*_max_) and maximal pressure variation during relaxation (−d*P*/d*t*_max_).

### Characterization of the crotonylome in rats with LPS-induced endotoxic shock

For crotonylproteomic profiling, myocardial tissues were collected from both the control and LPS groups. All tissue processing and subsequent proteomic analyses were conducted by investigators who remained blinded to the experimental group allocations. As shown in Fig. 2a, the samples were sonicated on ice using a high-intensity ultrasonic processor (Scientz) in lysis buffer. The remaining debris was removed by centrifugation at 12,000 × *g* at 4 °C for 10 min. Finally, the supernatant was collected, and the protein concentration was determined with a BCA kit (Beyotime) according to the manufacturer’s instructions. The whole-cell lysate was subsequently digested with trypsin (Promega) and labeled using one of the tandem mass tag 8-plex reagents. Crotonylated peptides were subsequently isolated through immunoaffinity enrichment with an anti-Kcr antibody. All the samples were combined in equal amounts and analyzed using liquid chromatography–tandem mass spectrometry (LC‒MS/MS). In brief, the tryptic peptides were dissolved in solvent A (0.1% formic acid, 2% acetonitrile/in water) and separated at a constant flow rate of 450 nl/min on a NanoElute ultrahigh-performance liquid chromatography (UHPLC) system (Bruker Daltonics). The separated peptides from the UHPLC system were then subjected to capillary source followed by the timsTOF Pro (Bruker Daltonics) mass spectrometry. The electrospray voltage applied was 1.65 kV. Precursors and fragments were analyzed at the time-of-flight (TOF) detector, with an MS/MS scan range from 100 to 1700 *m*/*z*. The timsTOF Pro was operated in parallel accumulation serial fragmentation (PASEF) mode.

A detailed analysis of the proteomics results is provided in the Supplementary Information, which includes data normalization, differential expression analysis and enrichment analysis.

### Histology analyses

Myocardial tissues were fixed in 4% paraformaldehyde, embedded in paraffin and then sectioned (5 µm). The tissue slides were then stained with hematoxylin‒eosin (H&E; Sangon Biotech) as previously described^27^. The lipids deposited in myocardial tissues were assessed using Oil Red O staining. In brief, myocardial tissues were fixed in 4% paraformaldehyde, embedded in optimal cutting temperature (OCT), sectioned into 10-μm-thick sections and then stained with Oil Red O (Sigma-Aldrich). All histological analysis of myocardial injury was performed by two independent pathologists who were blinded to the animal group identity.

### Transmission electron microscopy

The fixed myocardial tissues were rinsed with 0.1 M phosphate buffer (PB) three times (15 min each) and then fixed with 1% osmium tetroxide (O_S_O_4_; Ted Pella) in 0.1 M PB for 2 h at room temperature. Thereafter, the tissues were rinsed with 0.1 M PB three times (15 min each). After dehydration using a graded ethanol series (30%–50%–70%–80%–95%–100%–100%, 20 min each) and 100% acetone (twice, 15 min each), the tissues were embedded in resin. Ultrathin sections (60–80 nanoscale) were cut and then observed with an H-7650 Hitachi transmission electron microscope. To prevent observational bias, all sample processing, instrument operation and image acquisition were performed by a technician who was blind to the experimental conditions.

### Cell culture, adenovirus infection, and siRNA and plasmid transfection

The H9C2 rat cardiomyoblast cell line was purchased from Cellverse and cultured in Dulbecco’s modified Eagle medium iCell-128-0001, Cellverse) supplemented with 10% fetal bovine serum. Rat primary cardiomyocytes were purchased from Cellverse and cultured in rat primary cardiomyocyte culture medium (iCell-c001-002r, Cellverse). To model endotoxic shock-induced secondary cardiomyopathy, the cells were treated with 10 μg/ml LPS for 6 h.

#### Rat primary cardiomyocytes infected with recombinant adenoviruses

Recombinant adenoviruses for gene knockdown and overexpression were constructed for the infection of primary rat cardiomyocytes. For knockdown, short hairpin RNA sequences targeting rat CPT1B (5′-CGGTCTTGGTAGATGCTAA-3′), CBP (5′-GATGATGGAAGAGGATTTA-3′) and P300 (5′-AAGCGGCCTAAACTCTCATCTCC-3′), along with a nontargeting control (shNC, 5′-TTCTCCGAACGTGTCACGT-3′) were synthesized, cloned and inserted into the BglII/SalI site of the pShuttle-CMV shuttle vector (BR009, Hunan Fenghui Biotechnology) by Wuxi Genecefepharm Biotech. For overexpression, cDNA sequences encoding C-terminal Flag-tagged wild-type CPT1B (Flag-CPT1B-WT) or the K321R mutant (Flag-CPT1B K321R) were inserted similarly, with an empty vector (Ad-Vector) used as a control. Linearized shuttle vectors were cotransformed with the adenoviral backbone plasmid pAdEasy-1 into BJ5183-AD-1 electrocompetent *Escherichia coli* cells (Beijing Huayueyang Biotechnology) to generate full-length adenoviral genomes via homologous recombination. The resulting recombinant plasmids were linearized with PacI (Thermo Fisher Scientific) and transfected into HEK-293A (Cellverse) cells using Lipofectamine 3000 (Thermo Fisher Scientific) for virus production. Viruses were collected by repeated freeze‒thaw cycles and filtered through a 0.45-μm filter, then amplified to final titers of Ad-shNC at 2.3 × 10^9^ plaque-forming units (PFU)/ml, Ad-shCPT1B at 2.1 × 10^9^ PFU/ml, Ad-shCBP at 1.9 × 10^9^ PFU/ml, Ad-shP300 at 2.2 × 10^9^ PFU/ml, Ad-Vector at 2.4 × 10^9^ PFU/ml, Ad-Flag-CPT1B-WT at 1.4 × 10^9^ PFU/ml and Ad-Flag-CPT1B K321R at 1.2 × 10^9^ PFU/ml.

#### Plasmid transfection of H9C2 cells

To silence the P300 and CBP genes in H9C2 cells, the small interfering (si)RNAs used in this study were designed and synthesized by JTS Biotechnology. Specifically, si-NC (sense, 5′-UUCUCCGAACGUGUCACGUTT-3′; antisense, 5′-ACGUGACACGUUCGGAGAATT-3′) was used as a control for all the si-RNA experiments. si-P300 (sense, 5′-AAGCGGCCUAAACUCUCAUCUCCTT-3′; antisense, 5′-GGAGAUGAGAGUUUAGGCCGCUUTT-3′) and si-CBP (sense, 5′-GAUGAUGGAAGAGGAUUUATT-3′; antisense, 5′-UAAAUCCUCUUCCAUCAUCTT-3′). For plasmid transfection, H9C2 cells were transfected with a pcDNA3.1 plasmid carrying the C-terminal Flag-tagged CPT1B-WT (Flag-CPT1B-WT) or CPT1B K321R (Flag-CPT1B K321R) gene using Lipofectamine 3000 (Thermo Fisher Scientific) according to the manufacturer’s instructions. The referred plasmids were designed and synthesized by General Biol.

### ATP production assay

The ATP content in myocardial tissues and the indicated cell lines was measured using the ATP Assay Kit (Beyotime) according to the manufacturer’s instructions. In brief, ATP testing reagent (100 μl) was added to each well, along with 20-μl samples or reference standards. Two seconds later, ATP contents were measured as ATP/protein concentrations (nmol/mg protein). Data acquisition was carried out by technicians who were kept unaware of the experimental conditions associated with each sample.

### CPT1B protein activity assay

Cells were treated with 10 μg/ml LPS for 6 h and were assayed for CPT1B activity using a Carnitine Palmitoyl-Transferase I Assay Kit (Genmed Pharmaceutical Technology) according to the instructions of the manufacturer. Data acquisition was carried out by technicians who were kept unaware of the experimental conditions associated with each sample.

### BODIPY™ 493/503 fluorescence of LDs

To determine the lipid droplet (LD) distribution of in cells, the indicated cells were incubated with 20 μM BODIPY 493/503 (Shanghai Maokang Biotechnology) for 10 min. BODIPY 493/503 fluorescence was detected by laser confocal microscopy (Nikon A1+). To prevent observational bias, all sample processing, instrument operation and image acquisition were performed by a technician who was blind to the experimental conditions.

### Detection of the mitochondrial membrane potential (∆*Ψ*_m_)

As previously described^28^, the change in ∆*Ψ*_m_ in the indicated cells was detected with a Mitochondrial Membrane Potential Detection Kit (JC-1, Biosharp) according to the instructions. In brief, rat primary cardiomyocytes and H9C2 cells were incubated with JC-1 stock solution at 37 °C for 20 min. Thereafter, the cell samples were washed twice with JC-1 stock solution (1×). After centrifugation and resuspension, the cells were subjected to flow cytometry (NovoCyte, Agilent). Data acquisition was carried out by technicians who were kept unaware of the experimental conditions associated with each sample.

### Western blot and co-IP experiments

In accordance with the manufacturer’s instructions, myocardial tissues or the indicated cells were lysed in cell lysis buffer for western blot analysis and immunoprecipitation (IP; Beyotime) experiments on ice for 5 min, after which the lysates were centrifuged at 10,000*g* for 5 min. The concentration of total protein was determined using a BCA assay kit (Beyotime). Equal amounts of protein were separated by SDS‒PAGE and transferred onto polyvinylidene difluoride membranes (Abcam). After blocking with QuickBlock TBSTw buffer (Beyotime), the polyvinylidene difluoride membranes were then incubated with anti-CPT1B (no. 22170-1-AP; 1:500; Proteintech Group), anti-crotonyl-CPT1B K321 rabbit pAb (no. CA788O0305P1D-ET3; 1:1000; PTM-Biolab) or anti-α-tubulin (AC007; 1:1000; ABclonal) primary antibodies overnight at 4 °C. Afterward, the membranes were washed three times with western washing solution for 5 min each time. The membranes were subsequently incubated with HRP-labeled goat anti-rabbit IgG (A0208; 1:5000; Beyotime) at 37 °C for 45 min. The antigen‒antibody complex was detected using the BeyoECL Plus Assay (Beyotime). The amount of target protein was analyzed with Gel-Pro-Analyzer software. α-Tubulin expression was used for normalization. The M5 HiClear Prestained Protein Ladder (Mei5bio) was used as a visual reference during western blotting.

For the co-IP assay, the samples were lysed in cell lysis buffer for western blotting and IP (Beyotime), and the target proteins were purified with a Co-IP Assay Kit (Thermo Fisher Scientific) followed by western blotting. The following antibodies were used for western blotting: anti-crotonyl-CPT1B K321 rabbit pAb (no. CA788O0305P1D-ET3; 1:1000; PTM-Biolab), anti-pan-Kcr (PTM-501; 1:1000; PTM-Biolab), anti-Flag (AE063; 1:1000; ABclonal), anti-CPT1B (no. 22170-1-AP; 1:500; Proteintech Group), anti-P300 (sc-48343; 1:300; Santa Cruz Biotechnology) and anti-CBP (7389; 1:1000; Cell Signaling Technology). Band intensity quantification was conducted by investigators who remained blinded to the sample group assignments.

### Quantification of CPT1B K321 crotonylation using a site-specific antibody

In this study, for the specific quantification of CPT1B K321 crotonylation, a site-specific antibody (anti-crotonyl-CPT1B K321 rabbit pAb, no. CA788O0305P1D-ET3) against this modification was custom-generated through PTM-Biolab. The quality control data for this antibody can be found in the Supplementary Information.

### IP and LC‒MS/MS Identification

Liquid chromatography was carried out on a RIGOL L-3000 UPLC system (Rigol Technologies). For protein analyses, the initial flow conditions were 92% solvent A (H_2_O, containing 0.1% formic acid) and 8% solvent B (80% acetonitrile and 0.1% formic acid). Solvent B was increased to 95% in the subsequent 45 min and continued for 15 min. Positive MS/MS was performed on a Q Exactive HF-X mass spectrometer using the following parameters: Nanospray Flex (NSI) ion source; ion spray voltage, 2.2 kV; source temperature, 320 °C; scan range (*m*/*z*), 350–1,500; primary MS resolution, 120,000 (200 *m*/*z*); AGC, 3 × 10^6^; secondary MS/MS resolution, 15,000 (200 *m*/*z*); AGC, 5 × 10^4^; and collision energy for peptide fragmentation, 27%. Acetonitrile and formic acid were purchased from J.T. Baker and Sigma-Aldrich, respectively. All the solvents were LC/MS grade. Data acquisition was carried out by technicians who were kept unaware of the experimental conditions associated with each sample.

### Databases and data deposition

The protein domains and three-dimensional (3D) structure of CPT1B were obtained from the InterPro Database (https://www.ebi.ac.uk/interpro/protein/reviewed/Q63704/) and the AlphaFold Protein Structure Database (https://alphafold.ebi.ac.uk/entry/Q63704). The potential interacting proteins of CPT1B were identified by the UniProtKB database (https://www.uniprot.org/). The 3D structures of CPT1B (AF-Q92523), CBP (AF-Q92793) and P300 (AF-Q09472) were obtained from the AlphaFold Protein Structure Database (https://alphafold.ebi.ac.uk/). Molecular docking analysis was performed on the GRAMM Docking Web (https://gramm.compbio.ku.edu/). An online GPS-PAIL2.0 database (http://pail.biocuckoo.org/) was used to predict the histone acetyltransferase (HAT)-specific acetylation sites of the CPT1B protein. All raw mass spectrometry files, processed search results and associated proteomics data have been deposited to the ProteomeXchange consortium via the iProX repository (https://www.iprox.cn) and are publicly available under accession number PXD061951.

### Statistics

Statistical analysis was performed with GraphPad Prism 9. All the data are presented as the mean ± standard deviation (s.d.). Unless otherwise stated, comparisons between two groups were made using an unpaired *t*-test, while comparisons among three or more groups were analyzed by one-way analysis of variance, followed by Tukey’s multiple comparisons test. A *P* value <0.05 was considered to indicate statistical significance. For the proteomic experiments, differential changes in protein Kcr sites between the control and LPS-induced endotoxic shock groups were assessed using two-tailed Student’s *t*-tests for each site. Kcr sites with *P* < 0.05 and a fold change (FC) >1.5 or <2/3 were considered significantly altered.

## Results

### LPS-induced endotoxic shock

**Fig. 1:**
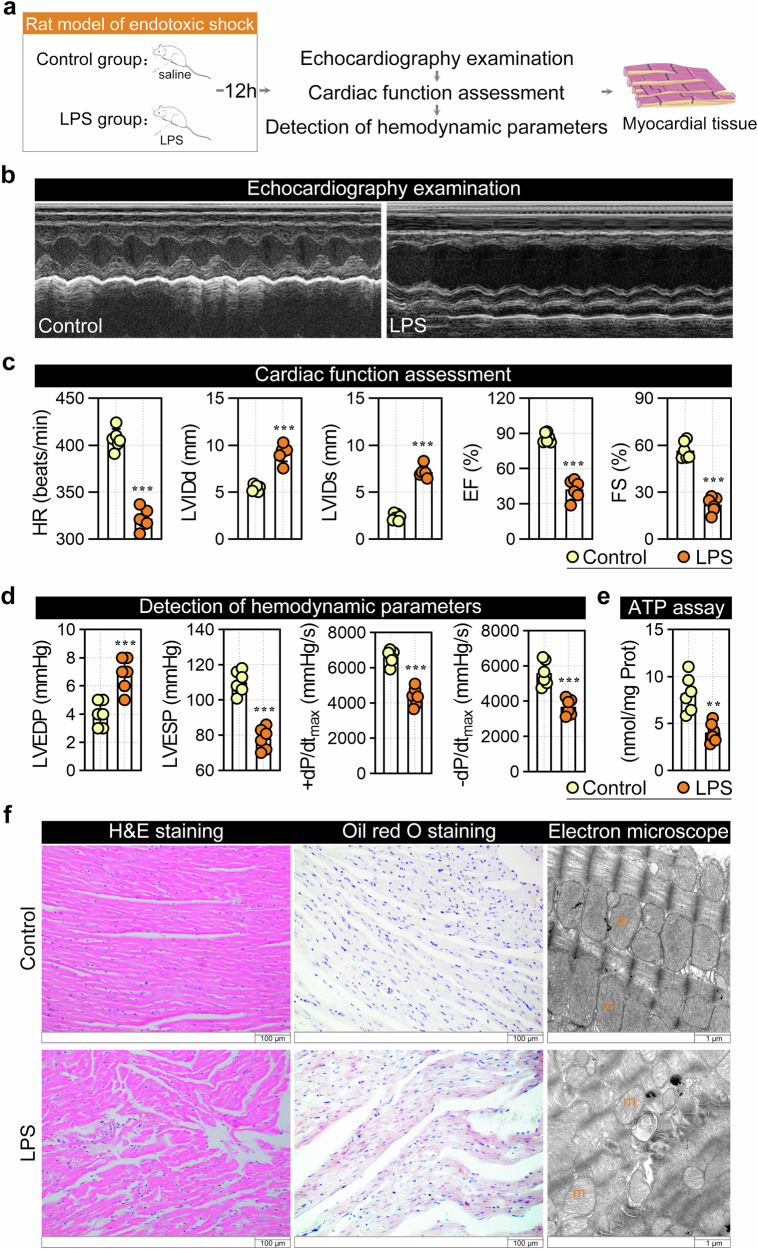
Establishment of the LPS-induced endotoxic shock rat model. **a** Experimental protocol. **b** Representative M-mode echocardiograms from LPS-treated and control rats. **c** Echocardiographic assessment of the HR, LVIDd, LVIDs, EF and FS. **d** Hemodynamic parameters including LVEDP, LVESP, maximal pressure variation during contraction (+d*P*/d*t*_max_) and maximal pressure variation during relaxation (−d*P*/d*t*_max_) were measured via left ventricular catheterization. **e** Myocardial ATP content was determined using the ATP Assay Kit. **f** Histological and ultrastructural analysis of myocardial tissues: H&E staining, Oil Red O staining for LDs, and transmission electron microscopy of myofibrils and mitochondria (m). Data are presented as mean ± s.d. **P < 0.01, ***P < 0.001 versus control group.

In line with our previous research^29^, a rat model of LPS-induced endotoxic shock was established to investigate endotoxic shock-induced secondary cardiomyopathy (Fig. 1a). As shown in Fig. 1b, we found that cardiac function was impaired in LPS-treated rats, as evidenced by significant decreases in HR, EF and FS and increases in LVIDd and LVIDs (Fig. 1c). The cardiotoxicity of LPS was also confirmed by abnormal hemodynamic parameters, such as increased LVEDP but decreased LVESP, +d*P*/d*t*_max_ and −d*P*/d*t*_max_ (Fig. 1d). Furthermore, ATP production was decreased in the myocardial tissues of the LPS-treated rats compared with those of the control rats (Fig. 1e), suggesting impaired mitochondrial function. Further transmission electron microscopy analysis also confirmed the disorder of the interfibrillar mitochondrial population, mitochondrial swelling and loss of the ridge structures (Fig. 1f). In addition, LPS-treated animals exhibited chaotic cardiac muscle fibers (Fig. 1f, H&E staining) and LD deposition (Fig. 1f, Oil Red O staining).

### Proteomic profiling of Kcr in myocardial tissue during LPS-induced endotoxic shock

**Fig. 2:**
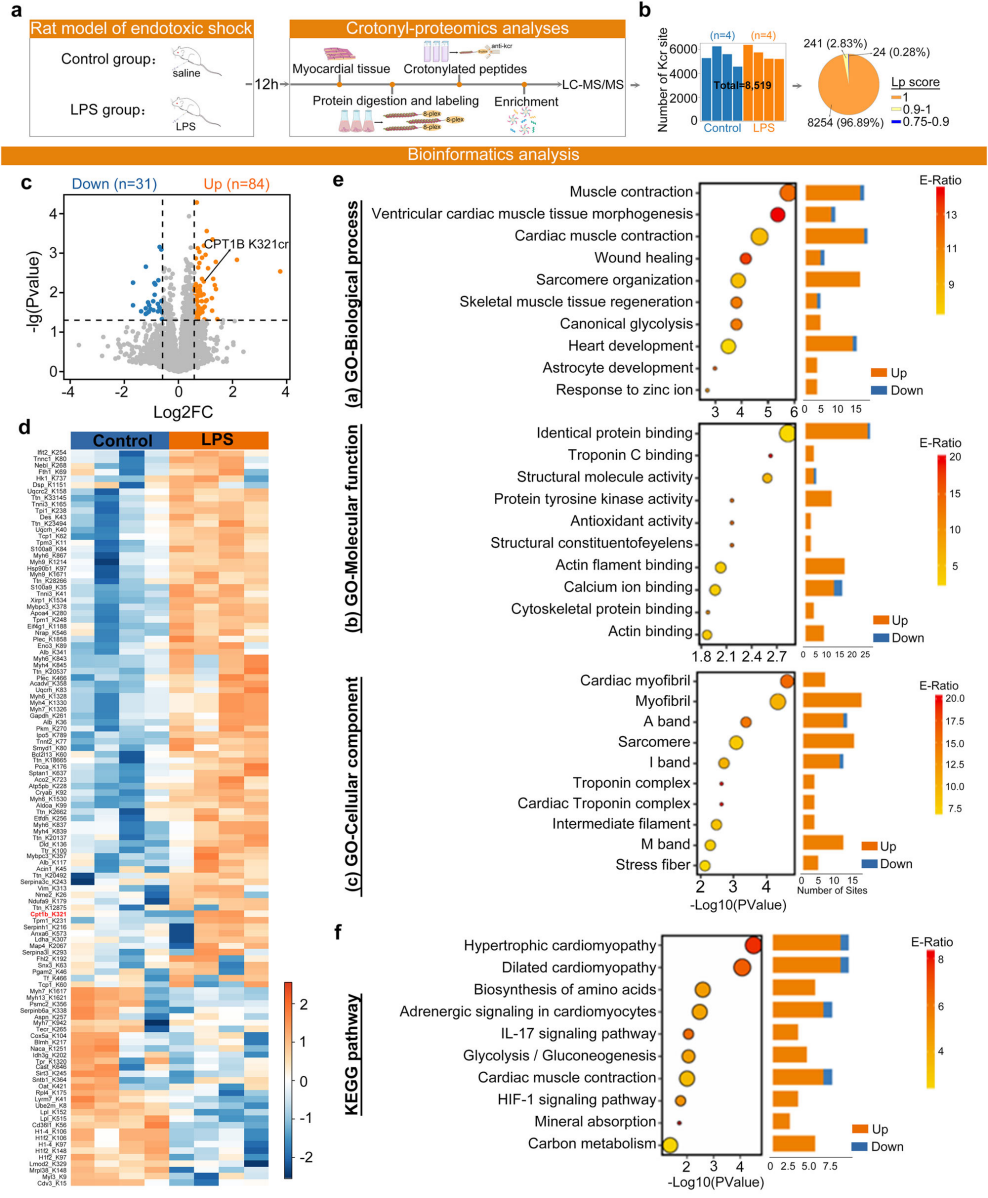
Crotonylproteomic profiling of myocardial tissues in a rat model of LPS-induced endotoxic shock. **a** Systematic workflow of the crotonylproteomic analysis. **b** Global characterization of cardiac Kcr modifications (left) and their LP scores (right). **c** Volcano plot of the differential Kcr sites (LPS group versus control group; cutoff: *P* value <0.05 and FC >1.5 or <2/3). **d** Heatmap of altered Kcr sites (orange, hypercrotonylation; blue, hypocrotonylation. **e,** **f** GO terms (**e**) and KEGG pathway enrichment analysis (**f**) for proteins with differential Kcr sites. Dot size, enriched protein count; bars, number of up-/downregulated Kcr sites.

For crotonylproteomic profiling, myocardial tissues were collected from the control and LPS groups. The whole-cell lysate of each sample was digested with trypsin and labeled using one of the tandem mass tag 8-plex reagents. Crotonylated peptides were isolated through immunoaffinity enrichment with an anti-Kcr antibody and subsequently analyzed by LC‒MS/MS (Fig. 2a). Overall, we identified a total of 8,519 Kcr sites, among which 8254 Kcr sites (96.9%) had a localization probability (LP) score of 1, where higher LP scores indicate a greater likelihood of accurate site localization (Fig. 2b). To ensure data reliability, we processed the raw MS/MS data, and identified 30,547 crotonylated peptides in the control group and 30,902 in the LPS group. Most peptides ranged from 7 to 20 amino acids (Supplementary Fig. 1a). The peptide length distribution met quality control standards. To maintain data quality, we retained 5,735 Kcr sites that were quantified in at least half of the samples (Supplementary Table 1). Principal component analysis demonstrated that the samples between the LPS group and the control group were distinct (Supplementary Fig. 1b). Next, to investigate crotonylome alterations associated with endotoxic shock, we identified 115 differentially expressed Kcr sites (FC >1.5 or <2/3, *P* < 0.05; Fig. 2c,d). Among these, 94.8% (109 sites) were detected on nonhistone proteins, while 6 sites were found on histones. Notably, 84 Kcr sites were upregulated and 31 were downregulated (Supplementary Table 2), suggesting a potential role for Kcr in the pathogenesis of LPS-induced endotoxic shock.

In addition, Gene Ontology (GO) and Kyoto Encyclopedia of Genes and Genomes (KEGG) enrichment analyses were performed on proteins with differential Kcr. Kcr-modified proteins were associated with cardiac muscle contraction and morphogenesis (Fig. 2e(a)), with enriched molecular functions including troponin C and actin binding (Fig. 2e(b)), indicating a direct role in regulating contractility. Cellular component analysis revealed that these proteins were localized to cardiac myofibrils, sarcomeric bands (A, I and M), and stress fibers (Fig. 2e(c)), supporting their involvement in maintaining structural integrity. KEGG analysis further revealed enrichment of cardiomyopathy-related pathways, cardiac muscle contraction and inflammatory signaling, such as the IL-17 pathway (Fig. 2f). Collectively, these results suggest that Kcr modifications may contribute to myocardial dysfunction by disrupting contractile protein interactions, compromising sarcomere stability and amplifying inflammatory responses, collectively driving structural remodeling and functional decline in endotoxic shock.

### K321 crotonylation (K321cr) of CPT1B in the LPS-induced endotoxic shock model

**Fig. 3:**
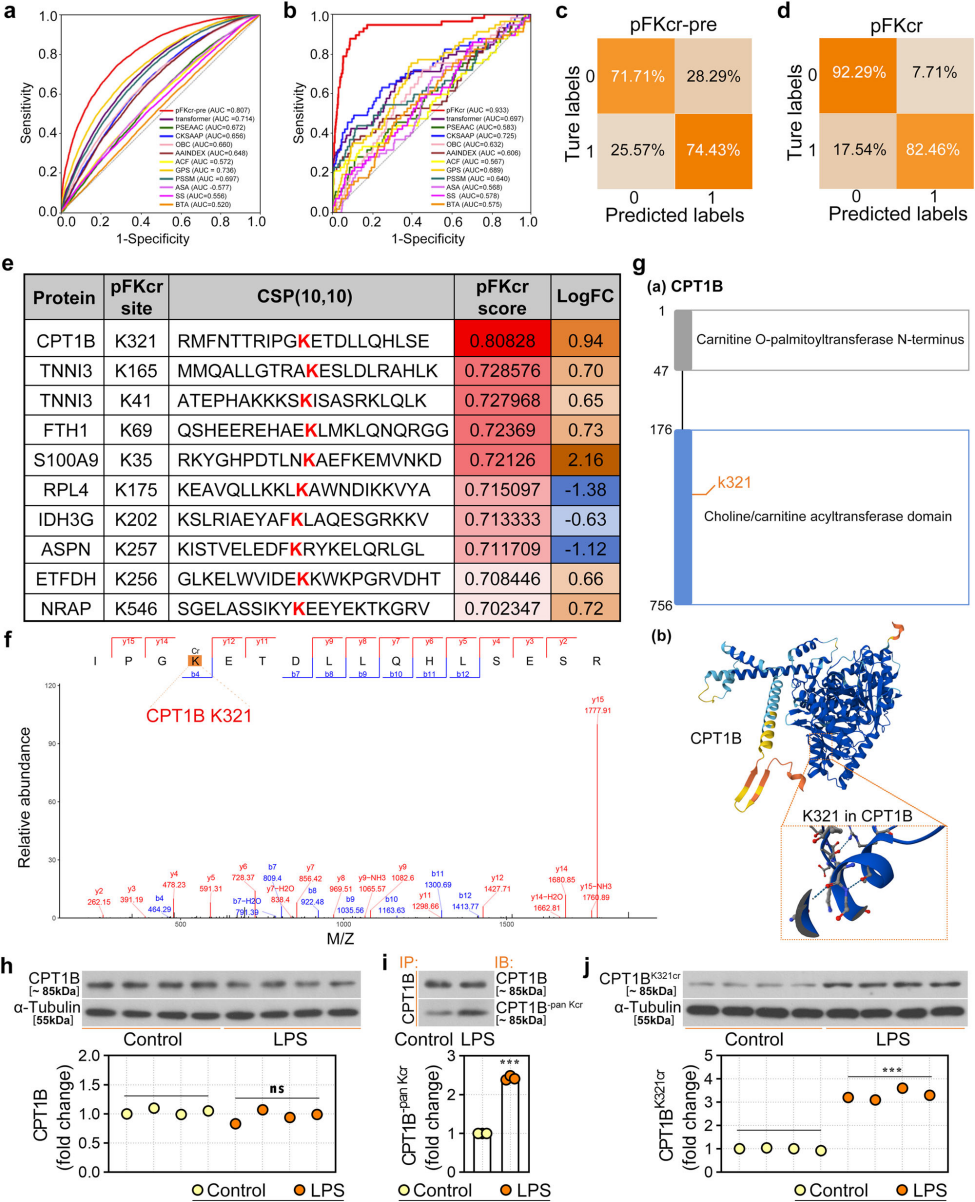
Prediction and experimental validation of CPT1B K321cr. **a**, **b** ROC curves and AUC values of prediction models pFKcr-pre (**a**) and pFKcr (**b**). **c**, **d** Confusion matrices for pFKcr-pre (**c**) and pFKcr (**d**) at a prediction threshold of 0.5. **e** Top ten predicted functional Kcr sites from pFKcr. **f** LC‒MS/MS identification of a candidate Kcr site (K321) on CPT1B. **g** Domain schematic and 3D structural localization of CPT1B K321: **g** (a) InterPro Database (https://www.ebi.ac.uk/interpro/protein/reviewed/Q63704/); (b) the AlphaFold Protein Structure Database (https://alphafold.ebi.ac.uk/entry/Q63704). **h** CPT1B protein expression levels were measured by western blot. **i** Co-IP validation of the pan-Kcr of CPT1B in myocardial tissues. **j** Western blot analysis of CPT1B K321cr in myocardial tissues. Data are presented as mean ± s.d. ***P < 0.001 versus control group; ns, not significant.

As shown in Supplementary Fig. 2a, the functional Kcr sites were predicted using the pFKcr framework, the detailed procedures of which are described in the Supplementary Information. The amino acid frequencies surrounding Kcr sites and functional Kcr sites in this dataset are shown in Supplementary Fig. 2b and Supplementary Fig. 2c, respectively. In addition, the receiver operating characteristic (ROC) curves and area under the curve (AUC) values for pFKcr-pre revealed that comparing pFKcr-pre with the 11 encoding schemes resulted in an improved performance with an AUC value ranging from 9.65% to 55.19% (Fig. 3a). For predicting functional Kcr sites, pFKcr had a high AUC value of 0.933 (Fig. 3b). The confusion matrices in Fig. 3c and Fig. 3d, evaluated at a threshold of 0.5, demonstrate that pFKcr-pre and pFKcr accurately identified Kcr sites and functional Kcr sites, respectively. Next, we identified the top ten highest-scoring Kcr sites, indicating functional importance (Fig. 3e). The most highly ranked functional Kcr site was CPT1B K321cr (score, 0.80828; logFC, 0.94), which was also identified by MS analysis (Fig. 3f). Structural analysis of the CPT1B gene revealed that the K321 site resides in the choline/carnitine acyltransferase domain, which constitutes its catalytic core^30^ (Fig. 3g(a)). The resolved crystal structure of the protein revealed the specific conformation of this region (Fig. 3g(b)). Compared with those in the control group, the expression levels of CPT1B protein in the myocardial tissues of the rats in the LPS group did not differ (Fig. 3h), but the pan-Kcr (Fig. 3i) and K321cr (Fig. 3j) levels in the LPS group significantly increased.

### LPS-mediated suppression of CPT1B activity in rat cardiomyocytes

**Fig. 4:**
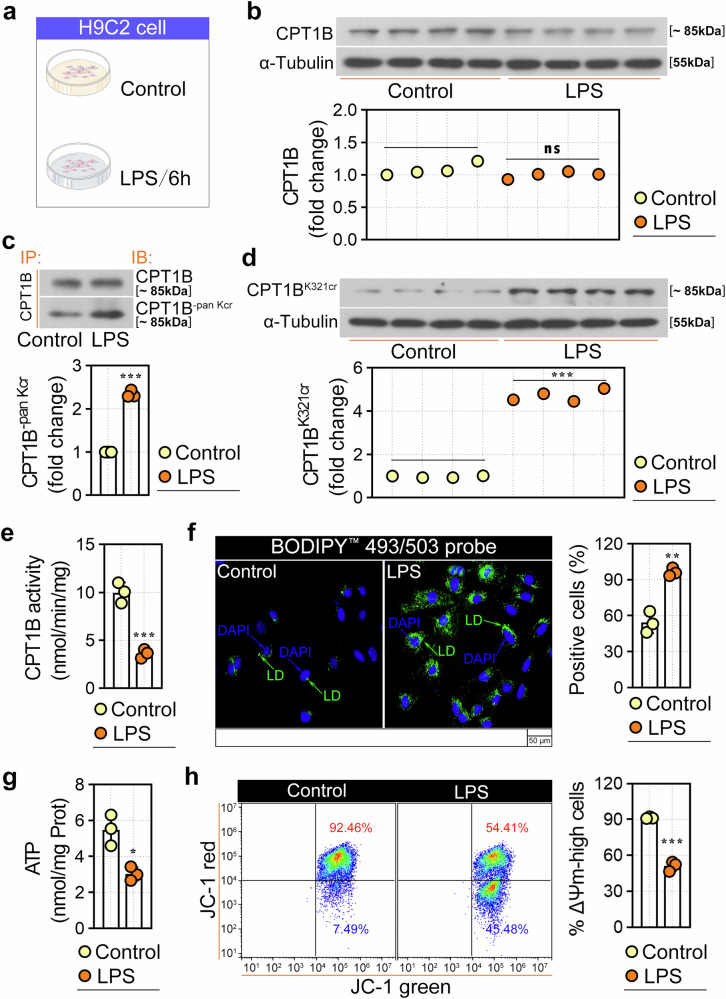
Effects of LPS on H9C2 cells. **a** H9C2 cells were treated with 10 μg/ml LPS for 6 h before the further assays. **b** CPT1B protein levels assessed by western blot. **c** Co-IP validation of the pan-Kcr of CPT1B. **d** Western blot analysis of CPT1B K321cr in H9C2 cells. **e** CPT1B enzymatic activity assay. **f** LD accumulation (BODIPY 493/503 staining) and quantification. **g** Cellular ATP content measurement. **h** Mitochondrial membrane potential (Δ*Ψ*_m_) assessed by JC-1 staining. Quantitative data are displayed in the adjacent bar chart. Data are presented as mean ± s.d. *P < 0.05, **P < 0.01, ***P < 0.001 versus control group; ns, not significant.

Furthermore, primary cardiomyocytes (Supplementary Fig. 3a) and H9C2 cells (Fig. 4a) were treated with LPS to induce myocardial cell injury. Consistent with the in vivo findings, LPS treatment did not affect the protein expression level of CPT1B in either H9C2 cells (Fig. 4b) or rat primary cardiomyocytes (Supplementary Fig. 3b). However, compared with those in the control group, the pan-Kcr (Fig. 4c and Supplementary Fig. 3c) and K321cr (Fig. 4d and Supplementary Fig. 3d) levels of CPT1B significantly increased following LPS treatment. Furthermore, we observed that CPT1B activity markedly decreased upon LPS treatment (Fig. 4e and Supplementary Fig. 3e). Moreover, BODIPY 493/503 fluorescence revealed LD deposition in LPS-treated H9C2 cells (Fig. 4f) and rat primary cardiomyocytes (Supplementary Fig. 3f). In addition, a reduction in ATP generation was evident in LPS-exposed H9C2 cells (Fig. 4g) and rat primary cardiomyocytes (Supplementary Fig. 3g), indicating that mitochondrial impairment was caused by LPS. This finding was further confirmed by the results of the JC-1 assay, which revealed a decrease in red fluorescence and an increase in green fluorescence after LPS treatment, indicating the loss of mitochondrial membrane potential (Fig. 4h).

### CPT1B K321 crotonylation mediated LPS-induced injury in rat cardiomyocytes

**Fig. 5:**
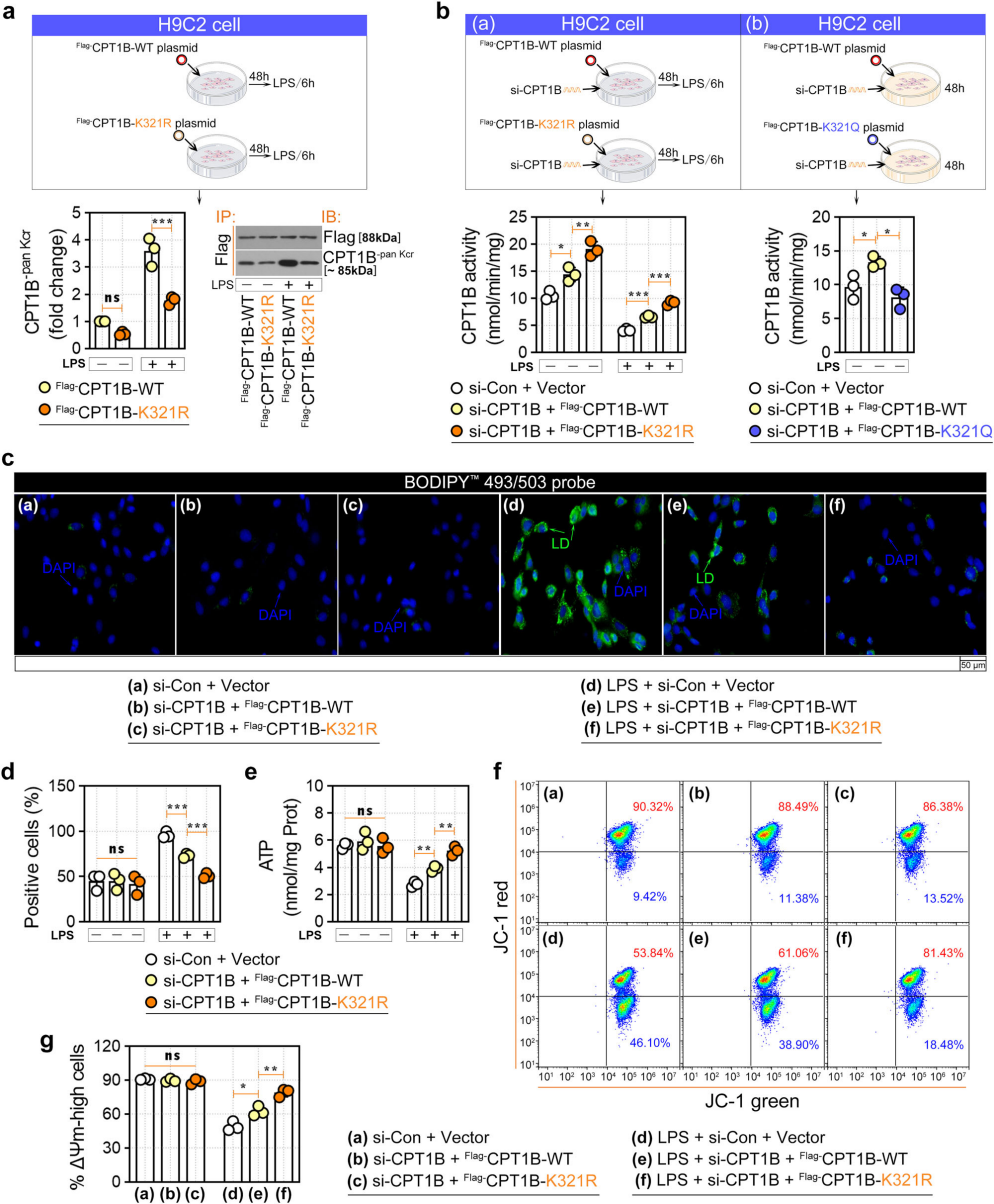
Functional necessity of CPT1B K321cr in LPS-induced H9C2 cell dysfunction. **a** H9C2 cells were transfected with Flag-tagged CPT1B-WT or -K321R mutant plasmids for 48 h, followed by 10 μg/ml LPS treatment. Co-IP validation of the pan-Kcr of exogenous CPT1B. **b** CPT1B enzymatic activity in H9C2 cells was measured under the following conditions: (a) cells were cotransfected with si-CPT1B and Flag-CPT1B-WT or Flag-CPT1B K321R mutant plasmids for 48 h, followed by treatment with or without 10 μg/ml LPS for 6 h; (b) cells were cotransfected with si-CPT1B and Flag-CPT1B-WT or Flag-CPT1B K321Q mutant plasmids for 48 h. **c** **d** LD accumulation (BODIPY 493/503 staining) (**c**) and quantification (**d**). **e** Cellular ATP content measurement. **f** Mitochondrial membrane potential (Δ*Ψ*_m_) assessed by JC-1 staining. **g** Quantitative data of Δ*Ψ*_m_ is displayed in adjacent bar chart. Data are presented as mean ± s.d. *P < 0.05, **P < 0.01, ***P < 0.001; ns, not significant.

To clarify the role of CPT1B K321cr, we mutated the K321 residue to arginine (R) to mimic the decrotonylation of CPT1B K321. As shown in Fig. 5a and Supplementary Fig. 4a, H9C2 cells and rat primary cardiomyocytes were transfected with the indicated plasmids or transduced with the indicated adenoviruses for 48 h. The K321R mutation itself did not significantly alter the pan-Kcr levels of CPT1B (Fig. 5a and Supplementary Fig. 4a). However, following LPS exposure, the K321R mutation led to a significant reduction in the pan-Kcr levels of CPT1B. These results indicated that K321 serves as the primary site for CPT1B crotonylation under conditions of LPS-induced cardiomyocyte injury. Next, H9C2 cells were transfected with si-CPT1B plasmids to eliminate interference from endogenous CPT1B (Fig. 5b), while primary rat cardiomyocytes were infected with adenovirus (Ad-shCPT1B) to achieve the same purpose (Supplementary Fig. 4b). We found that CPT1B activity increased upon the K321R mutation in both models (Fig. 5b(a) and Supplementary Fig. 4b), suggesting that this mutation prevents crotonylation of the protein by LPS. In addition, because the mutation of K321 to glutamic acid (Q) can roughly mimic the spatial structure of crotonylation^31^, the activity of CPT1B decreased upon K321-Q mutation even without LPS induction (Fig. 5b(b)). After excluding endogenous CPT1B interference, we further demonstrated that mutation of K321-R significantly alleviated LPS-induced LD deposition (Fig. 5c,d and Supplementary Fig. 4c) and mitochondrial dysfunction, as reflected by the recovery of ATP generation (Fig. 5e and Supplementary Fig. 4d) and mitochondrial membrane potential (Fig. 5f,g).

### Identification of the writers of CPT1B

**Fig. 6:**
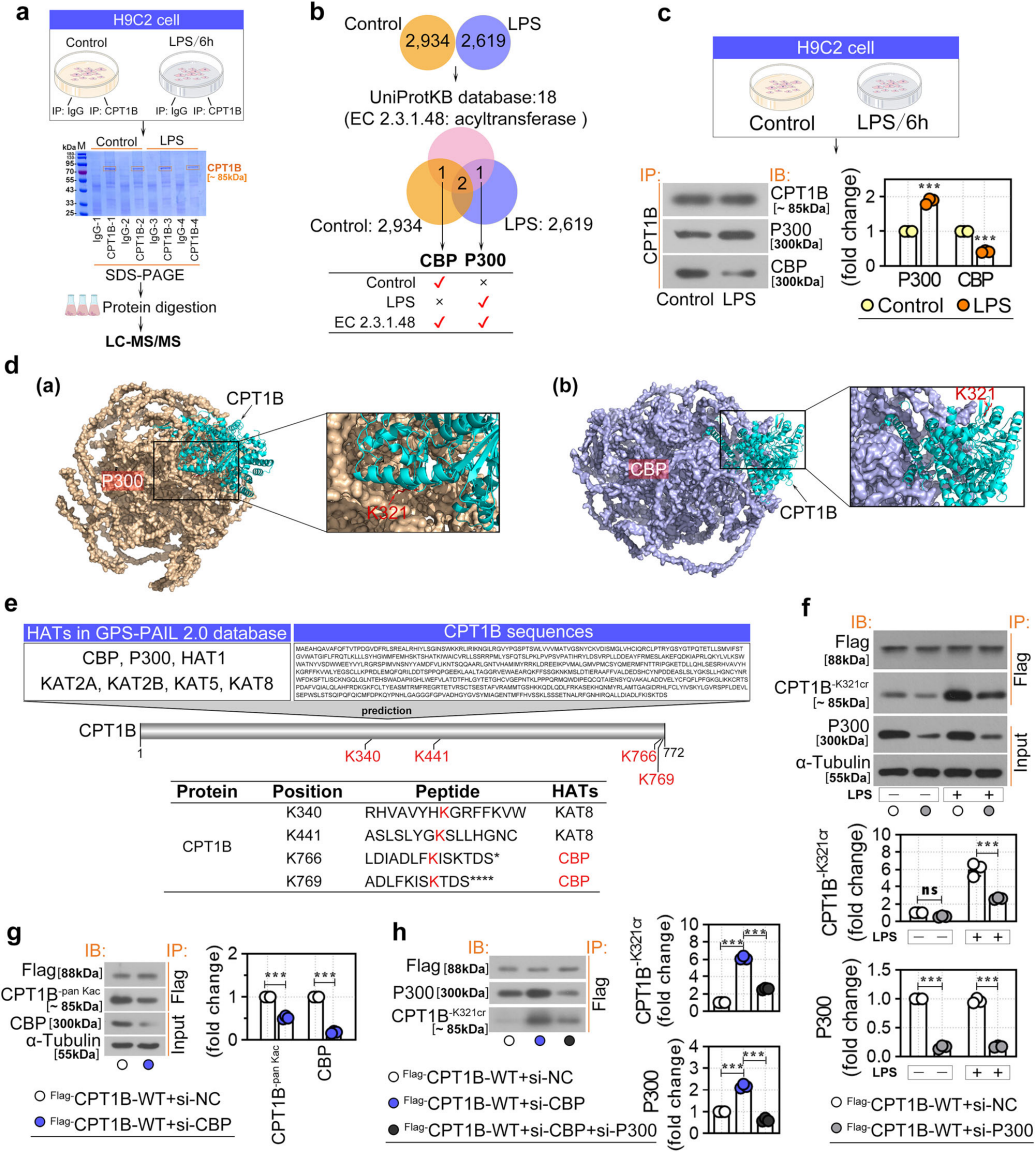
LC–MS/MS analysis of the interaction proteins of CPT1B in H9C2 cells. **a** Workflow for co-IP coupled with LC‒MS/MS analysis: H9C2 cells treated with 10 μg/ml LPS were subjected to co-IP with CPT1B antibody or IgG control. Coomassie blue staining confirmed CPT1B enrichment before LC–MS/MS analysis. **b** Venn diagrams of potential CPT1B interactors identified by LC–MS/MS versus known interactors in the UniProtKB database (https://www.uniprot.org/). **c** Co-IP validation of CPT1B interaction with CBP or P300 after LPS treatment. **d** Predicted binding interfaces between (a) CPT1B and P300 or (b) CBP: structures of CPT1B (AF-Q92523), P300 (AF-Q09472) and CBP (AF-Q92793) from the AlphaFold database (https://alphafold.ebi.ac.uk/); docking via GRAMM (https://gramm.compbio.ku.edu/). **e** The GPS-PAIL2.0 database (http://pail.biocuckoo.org/) was used to predict potential acetylation sites on CPT1B targeted by seven HATs (CBP, P300, HAT1, KAT2A, KAT2B, KAT5 and KAT8). **f** Cells cotransfected with Flag-CPT1B-WT and si-P300 were treated with or without LPS, followed by Flag-IP and immunoblotting for Flag, CPT1B K321cr and P300. **g** Cells cotransfected with Flag-CPT1B-WT and si-CBP were subjected to Flag-IP and immunoblotting for Flag, CPT1B^-pan Kac^ and CBP. **h** Cells cotransfected with Flag-CPT1B-WT, si-P300 and si-CBP were subjected to Flag-IP and immunoblotting for Flag, CPT1B K321cr and P300. Data are presented as mean ± s.d. ***P < 0.001; ns, not significant.

Because lysine crotonylation may disrupt the interaction of CPT1B with its binding partners, we immunoprecipitated H9C2 cells with anti-CPT1B with or without LPS, and the resulting pellets were eluted and analyzed by LC‒MS/MS (Fig. 6a) After the nonspecific proteins identified in the IgG group were excluded, 2,934 and 2,619 proteins that interacted with CPT1B were identified in the control and LPS-treated groups, respectively (Fig. 6b). To determine whether the binding proteins identified by LC‒MS/MS regulated the Kcr of CPT1B, we further analyzed acyltransferases (writer enzymes) from the UniProtKB database (EC2.3.1.48; https://www.uniprot.org/) to narrow the screening range. As shown in Fig. 6b, CBP was detected in both the control and EC2.3.1.48 groups but not in the LPS group. By contrast, P300 was detected in the LPS group but not in the control group. Consistently, the co-IP results confirmed that CPT1B binds to both CBP and P300 in H9C2 cells (Fig. 6c) and primary rat cardiomyocytes (Supplementary Fig. 5a). Moreover, in both cell models, CBP was released from its interaction with CPT1B in the LPS group, suggesting that the interaction between CPT1B and CBP might be impaired by LPS (Fig. 6c and Supplementary Fig. 5a). GO and KEGG enrichment analyses of the LC‒MS/MS-identified binding proteins revealed significant enrichment in terms related to lysine modification, N-terminal protein amino acid modification, acyltransferase activity (Supplementary Fig. 6a) and the cell cycle (Supplementary Fig. 6b). These findings highlight processes crucial for protein function regulation and cellular proliferation, suggesting their potential relevance to cardiomyocyte function in our model.

### LPS promoted P300-mediated CPT1B K321cr expression by inhibiting the binding of CBP and CPT1B

It has been reported that some acetyltransferases, such as CBP and P300 can catalyze both the Kac and Kcr of proteins^32^. To explore the specific effects of these two enzymes on the lysine modification of CPT1B, molecular docking analysis was performed on GRAMM Docking Web (https://gramm.compbio.ku.edu/). As shown in Fig. 6d, residue K321 of CPT1B is located at the interface between P300 and CPT1B (Fig. 6d(a)) but does not engage with CBP (Fig. 6d(b)). This structural positioning suggests that K321 is a potential site for selective crotonylation specifically by P300, but not by CBP. Furthermore, an online GPS-PAIL2.0 database (http://pail.biocuckoo.org/) was used to predict the HAT-specific acetylation sites of the CPT1B protein. Among the seven HATs (CBP, P300, HAT1, KAT2A, KAT2B, KAT5 and KAT8) recorded in this database, KAT8 and CBP were found to bind with CPT1B at K340, K441, K766 and K769, indicating that these two proteases might be involved in the Kac modification of CPT1B (Fig. 6e). On the basis of these predictive analyses, we hypothesized that P300, rather than CBP, might be involved in the K321cr of CPT1B.

To validate our hypothesis, co-IP assay was performed on the indicated H9C2 cells (Fig. 6f) and primary rat cardiomyocytes (Supplementary Fig. 5b). The results revealed that LPS treatment significantly increased CPT1B K321cr levels, whereas P300 silencing effectively restored the levels of CPT1B K321cr induced by LPS, suggesting that P300 mediates the effect of LPS on CPT1B K321cr (Fig. 6f and Supplementary Fig. 5b). Next, we found that knockdown of CBP significantly inhibited the pan-Kac levels of CPT1B (CPT1B^-pan Kac^), indicating that CBP occupied CPT1B in normal cells and increased its pan-Kac activity (Fig. 6g). Interestingly, in H9C2 cells, silencing CBP promoted K321cr in exogenous CPT1B, an effect that was reversed by concurrent P300 knockdown (Fig. 6h). Similar promotion of endogenous CPT1B K321cr expression was also observed in primary rat cardiomyocytes following CBP knockdown (Supplementary Fig. 5c). These findings suggest that the dissociation of CBP from CPT1B may facilitate the binding and crotonyl-transferase activity of P300, thereby increasing CPT1B K321cr levels.

### Mutation of K321 in CPT1B alleviated endotoxic shock-induced secondary cardiomyopathy in rats

**Fig. 7:**
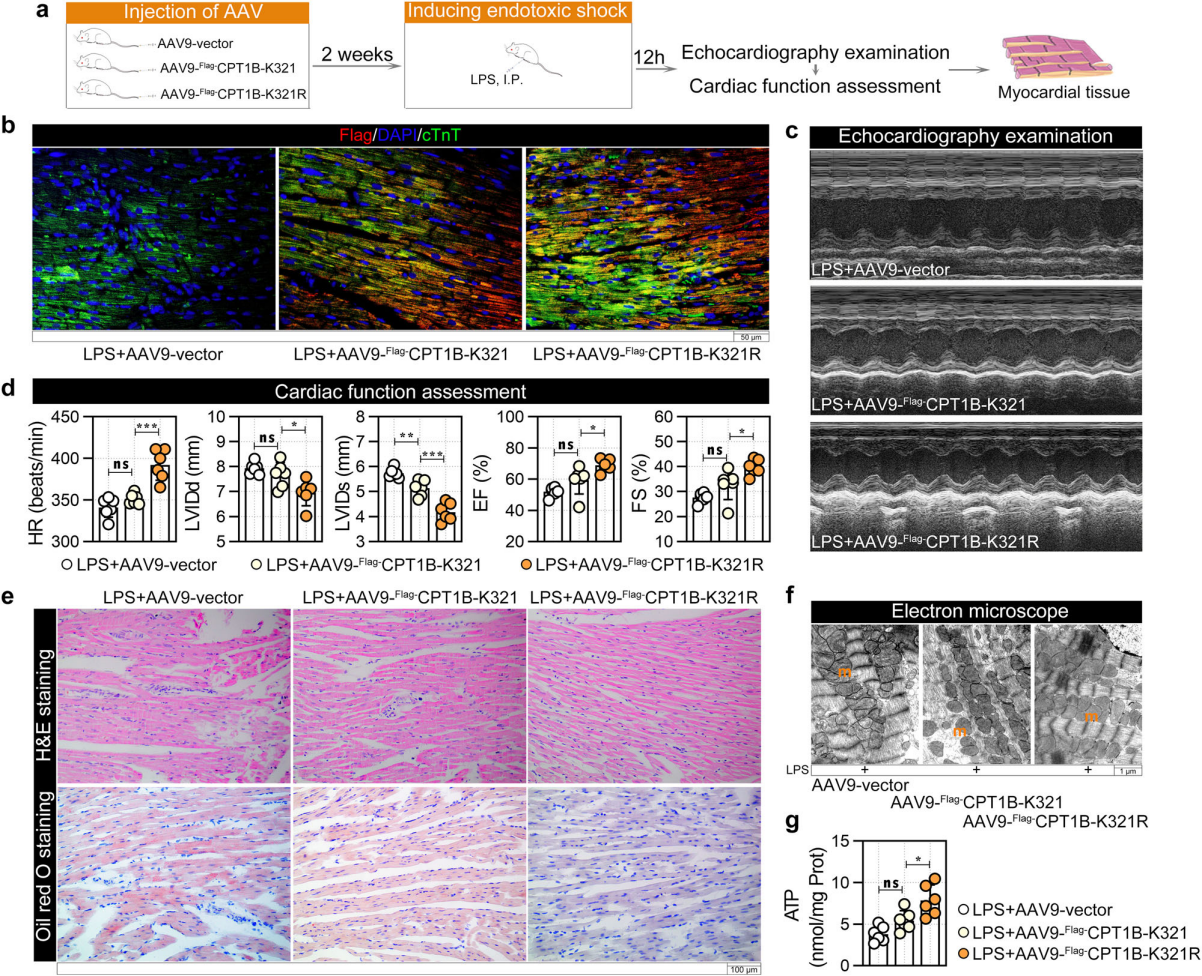
Effects of CPT1B K321cr on endotoxic shock-induced secondary cardiomyopathy in rats. **a** Experimental design for cardiac-specific AAV9 delivery followed by LPS-induced endotoxic shock in rats. **b** Immunofluorescent staining with cTnT (green), Flag tag (red) and nuclei (4′,6-diamidino-2-phenylindole (DAPI), blue). **c** Representative M-mode echocardiograms. **d** Echocardiographic assessment of the HR, LVIDd, LVIDs, EF and FS. **e**, **f** Histological (**e**) and ultrastructural (**f**) analysis of myocardial tissues: H&E staining, Oil Red O staining for LDs, and transmission electron microscopy of myofibrils and mitochondria (m). **g** Myocardial ATP content was determined using ATP Assay Kit. Data are presented as mean ± s.d. *P < 0.05, **P < 0.01, ***P < 0.001; ns, not significant.

To evaluate the relationship between CPT1B and endotoxic shock-induced secondary cardiomyopathy in vivo, first cardiomyocyte-specific AAV9 vectors (AAV9-Flag-CPT1B K321 and AAV9-Flag-CPT1B K321R) were injected into normal rats via the tail vein to increase cardiac CPT1B expression (Supplementary Fig. 7a). Two weeks later, echocardiography examination (Supplementary Fig. 7b) and cardiac function assessment (Supplementary Fig. 7c) confirmed that AAV9 injection did not affect normal heart function. Two weeks after AAV9 injection, endotoxic shock was induced by LPS injection (Fig. 7a). As shown in Fig. 7b, immunofluorescence confirmed the increased expression of CPT1B K321 and its mutant CPT1B K321R in rat cardiomyocytes. Compared with that in the control group, the impaired cardiac function induced by LPS was rescued by the expression of the CPT1B K321R mutant, as indicated by significant increases in HR, EF and FS, and decreases in LVIDd and LVIDs (Fig. 7c, d). As expected, the CPT1B K321R mutation also alleviated LPS-induced myocardial injury (Fig. 7e, H&E staining) and LD deposition (Fig. 7e, Oil Red O staining). Transmission electron microscopy analysis confirmed that the CPT1B K321R mutation also reversed LPS-induced mitochondrial swelling and loss of the ridge structures (Fig. 7f). The increase in ATP production further supported the recovery of mitochondrial function (Fig. 7g).

## Discussion

The advancement of HPLC‒tandem MS/MS for PTM identification has revealed the association of Kcr modification with various diseases, yet its role in endotoxic shock remains underexplored. An intraperitoneal injection of LPS has been established as a classic model for studying endotoxic shock^4^. Here, our crotonylproteomic analysis of LPS-induced endotoxic shock rat hearts first revealed widespread Kcr, particularly on proteins linked to cardiac pathology. We focused on CPT1B, whose K321 Kcr was significantly upregulated. Using site-specific mutagenesis in vitro and in vivo, we elucidated the pathophysiological role of CPT1B K321 in endotoxic shock-induced secondary cardiomyopathy.

CPT1B is the major mitochondrial outer membrane isoform responsible for long-chain fatty acid oxidation and is a critical contributor to myocardial ATP production and is essential for cardiac energetics^33,34^. Previous studies have demonstrated that CPT1B deficiency exacerbates systolic dysfunction in hypertrophied hearts^35^, while CPT1B overexpression rescues impaired fatty acid oxidation in cardiomyocytes with mitochondrial defects^36^. Owing to the high demand for ATP in the heart, the dysregulation of fatty acid oxidation leads to mitochondrial dysfunction, lipid deposition and contractile impairment^37^. These phenotypes are often observed in the hearts of patients with septic shock^38^. Our study revealed a novel mechanism underlying this pathology: LPS induced K321cr of CPT1B, which suppressed its enzymatic activity. Consequently, we observed decreased ATP production, lipid accumulation and mitochondrial membrane potential disruption in cardiac tissue from the LPS-induced endotoxic shock model. Crucially, introducing a K321R mutation restored CPT1B activity and rescued LPS-induced metabolic and functional deficits. While these findings established a clear association between CPT1B K321cr and impaired fatty acid oxidation in endotoxic shock-induced cardiomyopathy, it should be noted that CPT1B K321cr may correlate with, rather than directly cause, the observed mitochondrial dysfunction. Further research is needed to determine whether the suppression of CPT1B activity is the primary mechanism driving metabolic disruption or a contributing factor within a broader pathological network.

The identification and characterization of specific ‘writer’ enzymes is fundamental to understanding the regulatory mechanisms of Kcr^12^. Here, our proteomic analysis revealed the acyltransferases P300 and CBP in CPT1B Kcr. While both are well-established histone crotonyl-transferases^14,39^, accumulating evidence indicates that they also regulate nonhistone protein crotonylation, such as by promoting liver cancer metastasis through the crotonylation of Septin2^40^. Interestingly, these writer enzymes exhibit functional overlap with Kac, and Kcr can competitively inhibit acetylation^41^. In this study, we observed a dynamic balance between P300 and CBP in regulating CPT1B pan-Kcr and pan-Kac. Specifically, the dissociation of CBP from CPT1B facilitated P300 binding, thereby increasing CPT1B K321cr expression in both rat primary cardiomyocytes and H9C2 cells. This reciprocal regulatory relationship elucidates a novel mechanism of posttranslational crosstalk in the regulation of cardiac metabolism.

A key limitation of this study is its reliance on cardiac-specific AAV9-mediated gene delivery to investigate CPT1B in endotoxic shock-induced cardiomyopathy. Future studies using a conditional transgenic animal model with a cardiomyocyte-specific promoter are necessary to establish the causal role of CPT1B K321cr and to evaluate its therapeutic potential. Moreover, the impact of sex as a biological variable on cardiac signal transduction is well established. For example, Camper-Kirby et al.^42^ demonstrated the sex-dependent activation of Akt (protein kinase B), with females exhibiting higher levels of nuclear phospho-Akt^473^ than males. Consequently, it will be critically important to extend our current findings on CPT1B K321cr to female models. Determining whether this regulatory mechanism contributes to endotoxic shock in a sex-specific manner remains an open question for future research.

In summary, this study identified the protein Kcr as a critical regulator of endotoxic shock-induced cardiomyopathy. We established that Kcr at K321 of CPT1B impaired its enzymatic activity, thereby aggravating cardiac dysfunction during LPS-induced endotoxic shock. Mechanistically, LPS disrupted the dynamic equilibrium between CBP and P300 binding to CPT1B in cardiomyocytes, promoting P300 association and consequently increasing CPT1B K321cr levels. These findings suggested that CPT1B is a potential therapeutic target for endotoxic shock.

## Supplementary information

Supplementary Information

Supplementary Tables 1 and 2

Source Data
