# Supplementary material for: CPT1B K321 crotonylation contributes to cardiac dysfunction in endotoxic shock
Source: Exp Mol Med. 2026 May 28;58(5):1674–87. doi: 10.1038/s12276-026-01730-2 (PMC13233815; doi:10.1038/s12276-026-01730-2)
Supplement: Supplementary file 1 — Supplementary Information [file 12276_2026_1730_MOESM1_ESM.pdf]

Supplementary Material

1. Analysis of Protein Crotonylation in Proteomics

1.1 Data filtering and normalization

Two experimental groups (Control and LPS-treated rats) were established, each group had 4 biological replicates. The normalization process commenced with a $\log_2$ transformation of the intensity values of lysine (K) crotonylation (Kcr) sites to stabilize variance across measurements. Subsequently, median centering was applied to normalize the Kcr sites using the following formula:

$$NAV_{ij} = log_2(Intensity_{ij}) - Median\left(log_2(Intensity_j)\right),$$

where i denotes the Kcr site and j represents the sample. This normalization step ensured that the normalized abundance values (NAVs) of the Kcr sites exhibited a centered logarithmic normal distribution around zero. Following normalization, Kcr sites with more than 50% missing intensity values across all samples were excluded to reduce noise and enhance data reliability. For the remaining missing intensity values, imputation was performed using the Perseus method. This method involves shrinking the distribution of detected values to a factor of 0.3 (width), shifting it down by 1.8 standard deviations (down shift), and simulating random values to fill in the missing data. This targeted imputation approach effectively addresses potential biases caused by missing data, resulting in a robust and reliable dataset for subsequent analyses.

1.2 Differential expression analysis

Following data filtering and normalization, we proceeded to identify differentially expressed Kcr sites between the two experimental groups (Control and LPS-treated rats). The analysis was conducted using the data prior to imputation to ensure the integrity of the statistical comparisons.

For each Kcr site, we calculated the mean $\log_2$ NAV for both the experimental and control groups. The log fold change (logFC) for each Kcr site was then determined by subtracting the mean $\log_2$ NAV of the control group from that of the experimental group:

$$logFC_i = Mean(log_2(Experiment\ Nav_i)) - Mean(log_2(Control\ Nav_i)),$$

where i denotes the Kcr site. To assess the statistical significance of these changes, we

performed two-tailed Student's t-tests for each Kcr site. Only Kcr sites with *P* value < 0.05 and FC > 1.5 or FC < 2/3 were considered significantly changed.

1.3 Enrichment analysis

To perform Gene Ontology (GO)-based enrichment analysis on proteins containing differentially Kcr sites, we utilized a two-sided hypergeometric test. The following parameters were defined for the analysis:

N = total number of crotonylated proteins annotated with at least one term

n = number of crotonylated proteins associated with a specific GO term *t*

M = total number of differentially crotonylated proteins (DCPs) annotated with at least one term

m = number of DCPs associated with the term *t*

Using these parameters, we calculated the enrichment ratio (E-ratio) and the corresponding p-value based on the hypergeometric distribution as follows:

$$\text{E-ratio} = \frac{\frac{m}{M}}{\frac{n}{N}}$$

$$p = \sum_{m'=m}^{n} \frac{\binom{M}{m'}\binom{N-M}{n-m'}}{\binom{N}{n}}, \ (\text{E-ratio} > 1)$$

GO annotation files, released on March 3, 2023, were obtained from the Gene Ontology Consortium website (http://www.geneontology.org/).

1.4 Pretraining and architecture of the framework named "Prediction of Functional Kcr Sites" (pFKcr)

We developed a four-layer DNN comprising two dense layers interspersed with two dropout layers to process eleven distinct features and generate scores for CSP (10, 10). The Rectified Linear Unit (ReLU) activation function was employed to introduce nonlinearity into the DNN, effectively addressing the vanishing gradient problem:

$$ReLU(x) = \begin{cases} x, & x \geq 0 \\ 0, & x < 0 \end{cases}$$

In the output layer, two neurons with sigmoid activation functions were utilized to compute the final score for each CSP (10,10) peptide y:

$$P_{Kcr}(y) = sigmoid(y) = \frac{1}{1 + e^{-y}}$$

Each of the eleven features was individually scored by the DNN for every CSP (10,10),

resulting in an 11-dimensional vector V = (D1, D2, D3, …, D11) containing these scores. Subsequently, we constructed an integrated four-layer DNN model that takes the 11-dimensional vector V as input to produce the final prediction score. The parameters for the DNNs corresponding to the features are detailed in the following **Table 1**.

Table 1 Summary of determined parameters in pFKcr framework for the Kcr sites and functional Kcr sites predictions.

**Models**	**Parameters**	**Values**
PseAAC	Input	20
	Hidden 1	2048
	Dropout 1	0.5
	Hidden 2	1024
	Dropout 2	0.5
	Output	2
	Pre-training epochs	50
	Transfer-learning epochs	91
CKSAAP	Input	441
	Hidden 1	2048
	Dropout 1	0.5
	Hidden 2	1024
	Dropout 2	0.5
	Output	2
	Pre-training epochs	50
	Transfer-learning epochs	70
OBC	Input	441
	Hidden 1	64
	Dropout 1	0.3
	Hidden 2	32
	Dropout 2	0.3
	Output	2
	Pre-training epochs	50
	Transfer-learning epochs	75
AAindex	Input	210
	Hidden 1	1024
	Dropout 1	0.1
	Hidden 2	512
	Dropout 2	0.1

Output	2
Pre-training epochs	50
Transfer-learning epochs	66

Continuation of Table 1

**Models**	**Parameters**	**Values**
ACF	Input	210
	Hidden 1	2048
	Dropout 1	0.1
	Hidden 2	1024
	Dropout 2	0.1
	Output	2
	Pre-training epochs	50
	Transfer-learning epochs	85
GPS	Input	300
	Hidden 1	512
	Dropout 1	0.2
	Hidden 2	256
	Dropout 2	0.2
	Output	2
	Pre-training epochs	50
	Transfer-learning epochs	75
PSSM	Input	420
	Hidden 1	1024
	Dropout 1	0.2
	Hidden 2	512
	Dropout 2	0.2
	Output	2
	Pre-training epochs	50
	Transfer-learning epochs	70
ASA	Input	21
	Hidden 1	2048
	Dropout 1	0.2
	Hidden 2	1024
	Dropout 2	0.2
	Output	2
	Pre-training epochs	50
	Transfer-learning epochs	45
SS	Input	63
	Hidden 1	2048
	Dropout 1	0.2
	Hidden 2	1024

Dropout 2	0.2
Output	2
Pre-training epochs	50
Transfer-learning epochs	45

Continuation of Table 1

**Models**	**Parameters**	**Values**
BTA	Input	84
	Hidden 1	2048
	Dropout 1	0.2
	Hidden 2	1024
	Dropout 2	0.2
	Output	2
	Pre-training epochs	50
	Transfer-learning epochs	80
Transformer	Input	128
	Hidden 1	2048
	Dropout 1	0.5
	Hidden 2	1024
	Dropout 2	0.5
	Output	2
	Pre-training epochs	50
	Transfer-learning epochs	62
Integrated_DNN	Input	11
	Hidden 1	1024
	Dropout 1	0.5
	Hidden 2	512
	Dropout 2	0.5
	Output	2
	Pre-training epochs	50

2. Quality Control for A Site-Specific Antibody Against CPT1B K321 Crotonylation

2.1 ELISA assay

In ELISA using a 96-well plate coated with the CPT1B K321cr peptides and a control (unmodified) peptide, the anti-crotonyl-CPT1B K321 Rabbit pAb was incubated at various dilutions (e.g., 1:54K, 1:162K). The results showed a positive signal (OD_{450} > 1.0) at antibody dilutions greater than 1:54,000, with a signal more than 10-fold higher than that for the unmodified control peptide (**Figure 1**).

Antibody / Coat / Dilution	Anti-crotonyl-CPT1B K321		
	CPT1B K321cr peptide A	CPT1B K321cr peptide B	Control unmodified peptide
1:2K	1.847	1.962	1.077
1:6K	2.027	1.956	0.491
1:18K	1.838	1.833	0.243
1:54K	1.411	1.489	0.117
1:162K	0.903	0.856	0.064
1:486K	0.453	0.387	0.063
1:1458K	0.191	0.192	0.535
1:4374K	0.048	0.05	0.051

Figure 1

2.2 Dot-Blot Assay

Dot blot analysis was performed by immobilizing varying amounts (e.g., 4 ng, 16 ng) of the CPT1B K321cr peptides and a control (unmodified) peptide on a solid-phase membrane. The membrane was then incubated with the anti-crotonyl-CPT1B K321 Rabbit pAb, followed by an enzyme-conjugated secondary antibody and chemiluminescent substrate. The results demonstrated that the antibody exhibited strong binding to the CPT1B K321cr peptides (**Figure 2**).

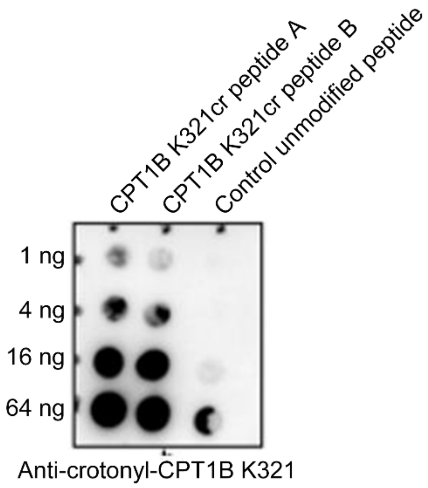

Figure 2

2.3 Western blot assay

The anti-crotonyl-CPT1B K321 Rabbit pAb was validated by Western blot using tissue lysates from rat heart and rat skeletal muscle. As shown in **Figure 3**, a band of approximately 85 kDa was detected in rat heart lysates, corresponding to the target protein $CPT1B^{K321cr}$. This result indicates that the antibody successfully recognizes endogenous $CPT1B^{K321cr}$, and the observed molecular weight is accurate.

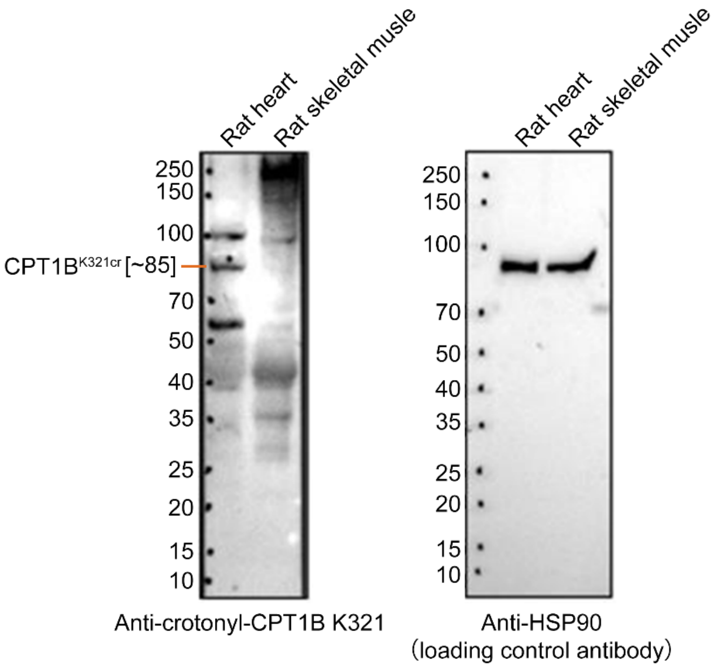

Figure 3

Supplementary figures

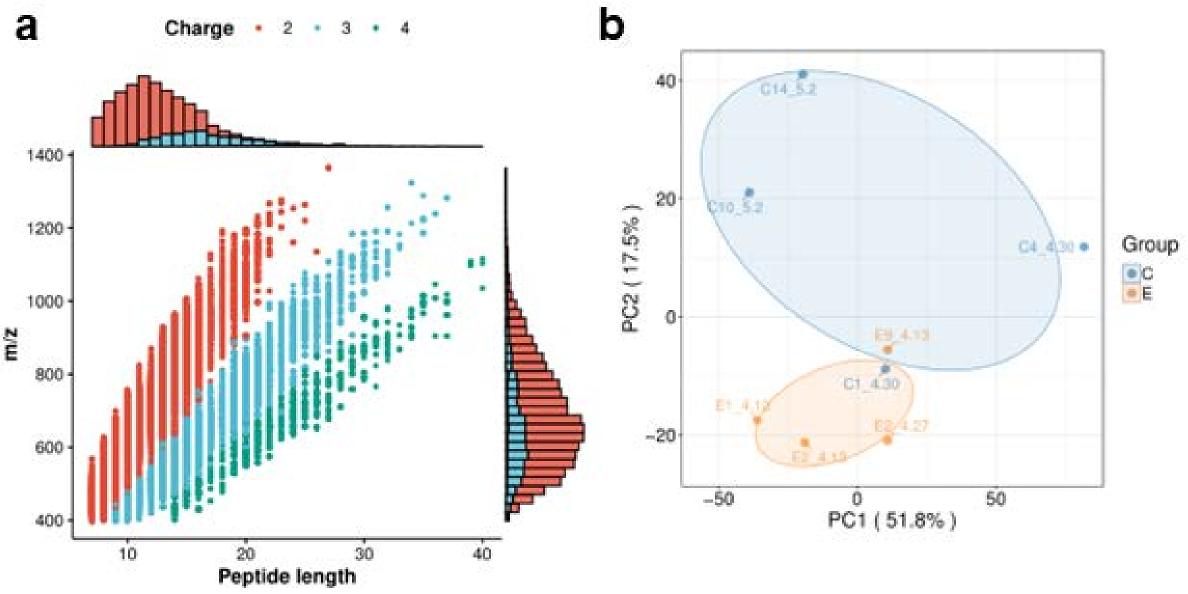

Supplementary Fig. 1 Quality control for crotonylproteomic analysis

(a) Distribution of peptides length. **(b)** Principal component analysis (PCA) of the crotonylome profiles, showing separation between LPS-treated (Group E) and control samples (Group C).

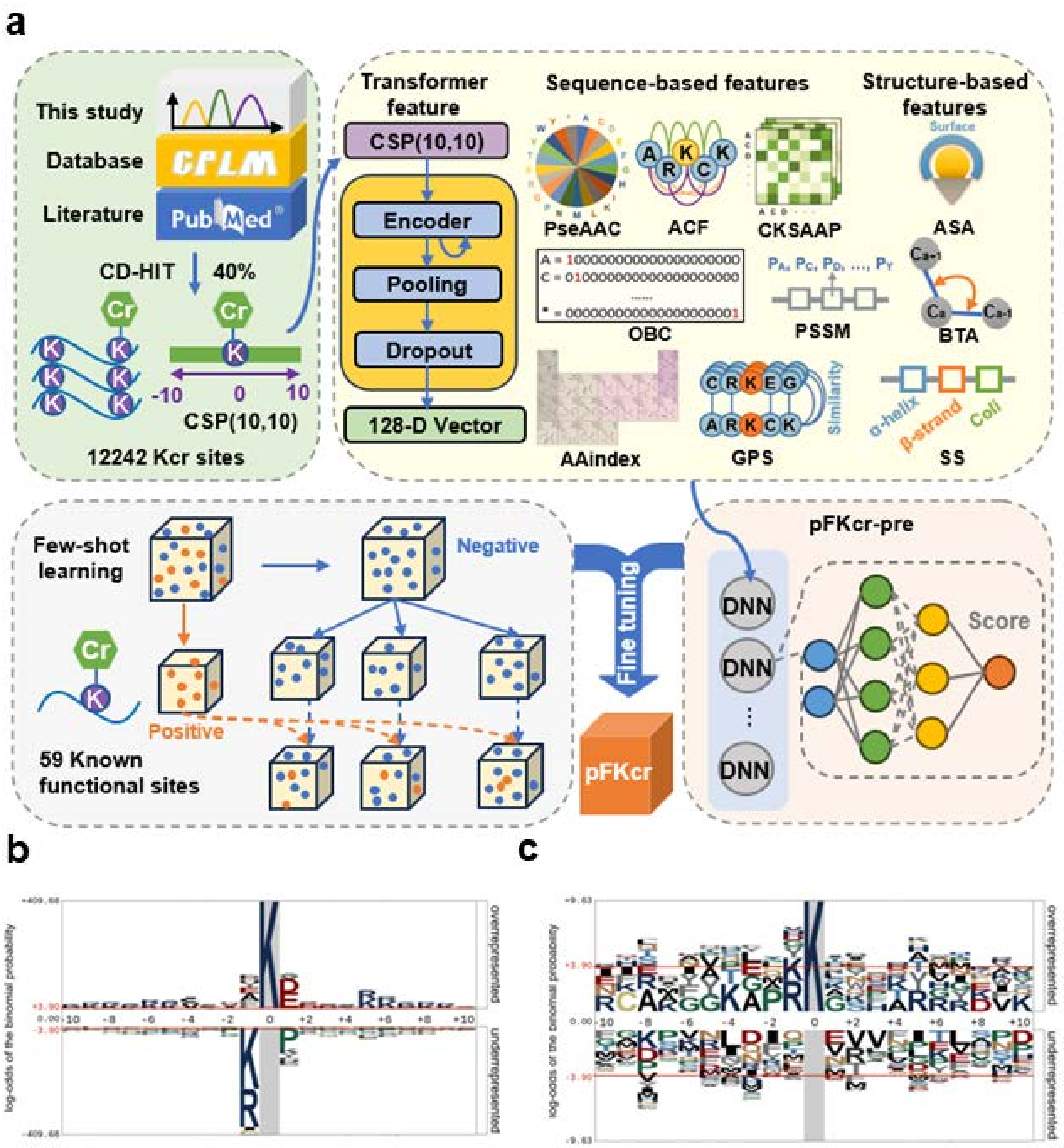

Supplementary Fig. 2 Hybrid protein language model (PLM)-based framework of “Prediction of Functional Kcr Sites” (pFKcr)

(a) Overview of the pFKcr-pre and pFKcr frameworks, integrating multi-feature hybrid learning, transformer-based PLM, and few-shot transfer learning strategies. **(b, c)** Amino acid frequencies surrounding (b) Kcr sites and (c) functional Kcr sites in this dataset.

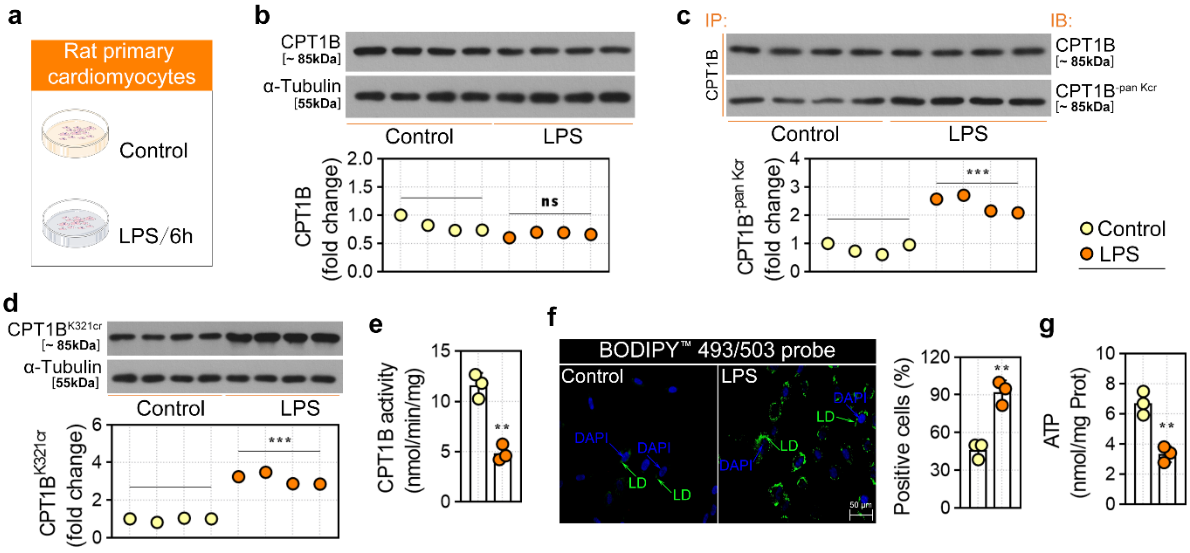

Supplementary Fig. 3 Effects of LPS on rat primary cardiomyocytes

(a) Rat primary cardiomyocytes were treated with 10 μg/mL LPS for 6 hours prior to the further assays. **(b)** CPT1B protein levels assessed by western blot. **(c)** Co-IP validation of the pan-Kcr of CPT1B. **(d)** Western blot analysis of CPT1B K321cr in primary cardiomyocytes. **(e)** CPT1B enzymatic activity assay. **(f)** Lipid droplet accumulation (BODIPY™ 493/503 staining) and quantification. **(g)** Cellular ATP content measurement. Data are presented as mean ± SD. $**p < 0.01$ and $***p < 0.001$ *vs.* control group; ns, not significance.

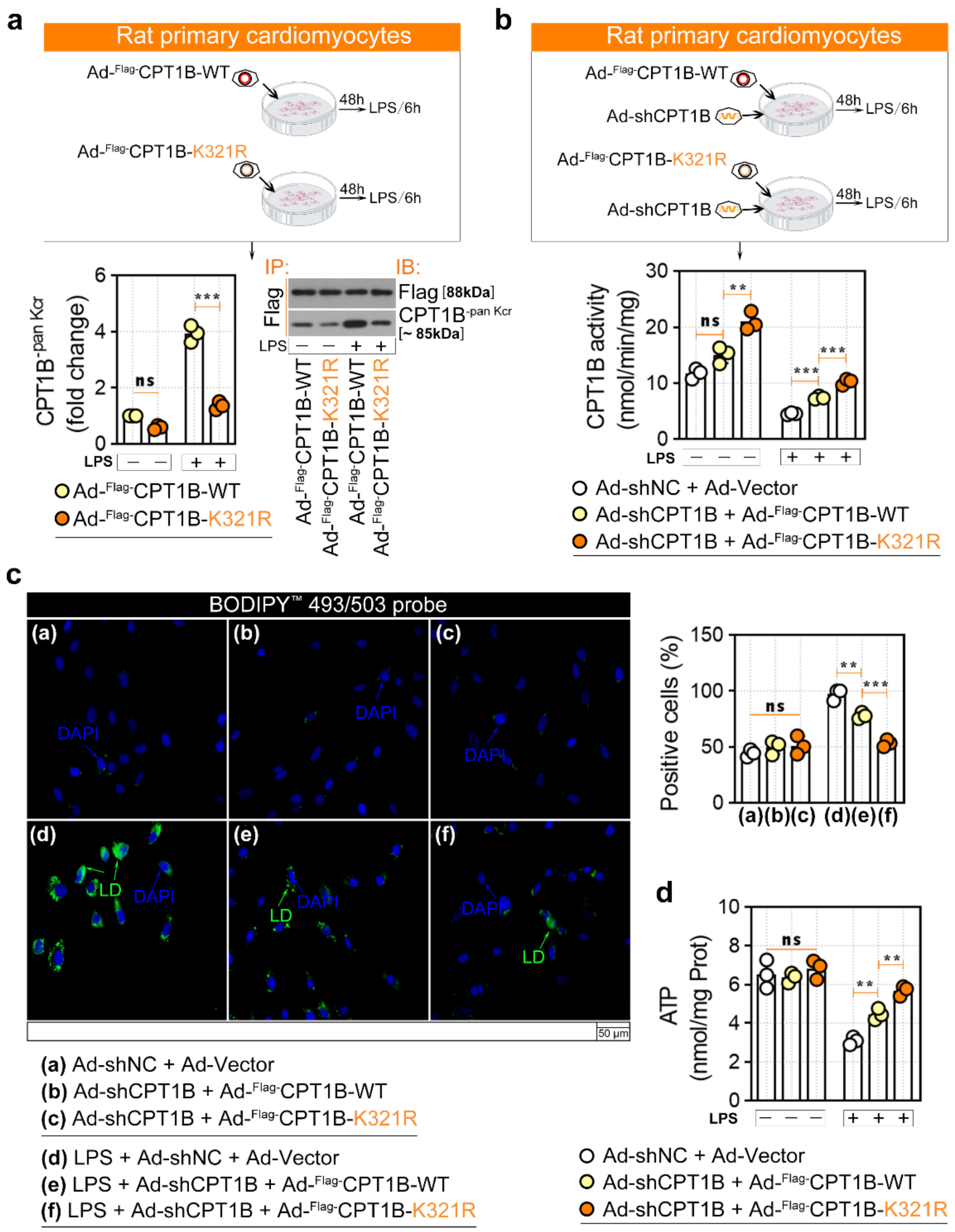

Supplementary Fig. 4 CPT1B K321cr is essential for LPS-induced dysfunction in rat primary cardiomyocytes

(a) Rat primary cardiomyocytes were infected with recombinant adenoviruses (Ad) carrying Flag-tagged CPT1B-WT or -K321R for 48 hours, followed by treatment with or without 10 μg/mL LPS for 6 hours, the pan-Kcr of exogenous CPT1B was validated by Co-IP. **(b)** After co-infection with Ad-shCPT1B and either Ad-^{Flag}-CPT1B-WT or -K321R, followed by

treatment with or without 10 μg/mL LPS for 6 hours, CPT1B enzymatic activity was measured in the cells. **(c)** Lipid droplet accumulation (BODIPY™ 493/503 staining) and quantification. **(d)** Cellular ATP content measurement. Data are presented as mean ± SD. $^{**}p < 0.01$ and $^{***}p < 0.001$; ns, not significance.

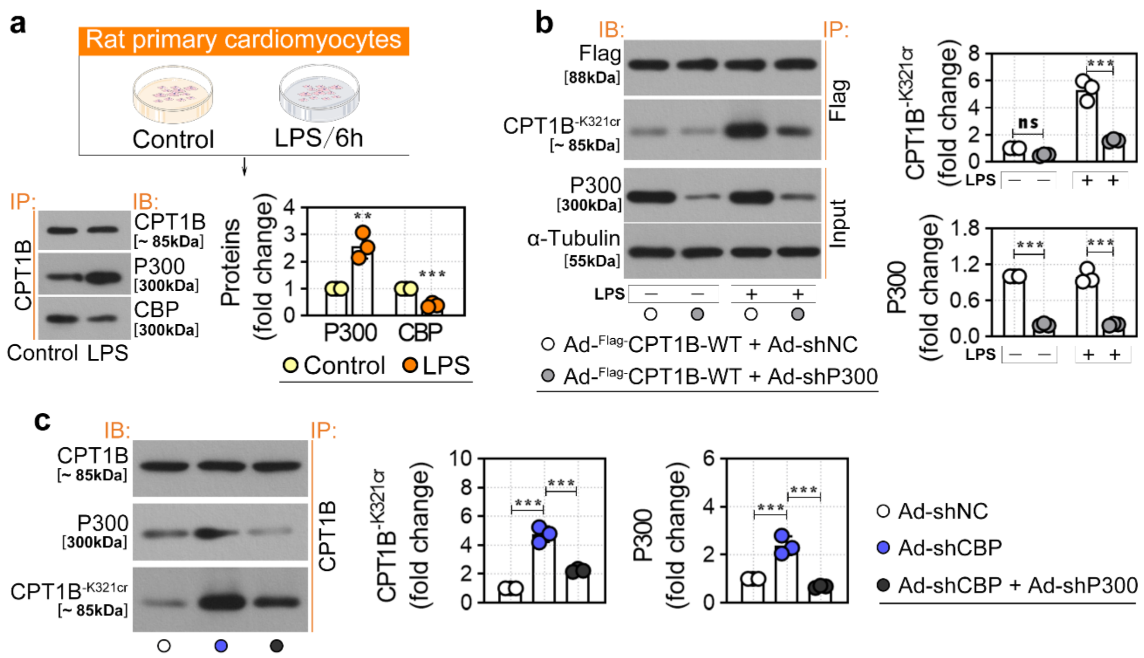

Supplementary Fig. 5 LPS promoted P300-mediated CPT1B-K321cr expression in rat primary cardiomyocytes

(a) Co-IP validation of CPT1B interaction with CBP or P300 after LPS treatment. **(b)** Cells co-infection with Ad-$^{Flag-}$CPT1B-WT and Ad-shP300 were treated with or without LPS, followed by Flag-IP and immunoblotting for Flag and CPT1B$^{-K321cr}$. **(c)** Cells co-infection with Ad-shCBP and Ad-shP300 were subjected to CPT1B-IP and immunoblotting for CPT1B, P300 and CPT1B$^{-K321cr}$. Data are presented as mean ± SD. $^{**}p < 0.01$ and $^{***}p < 0.001$; ns, not significance.

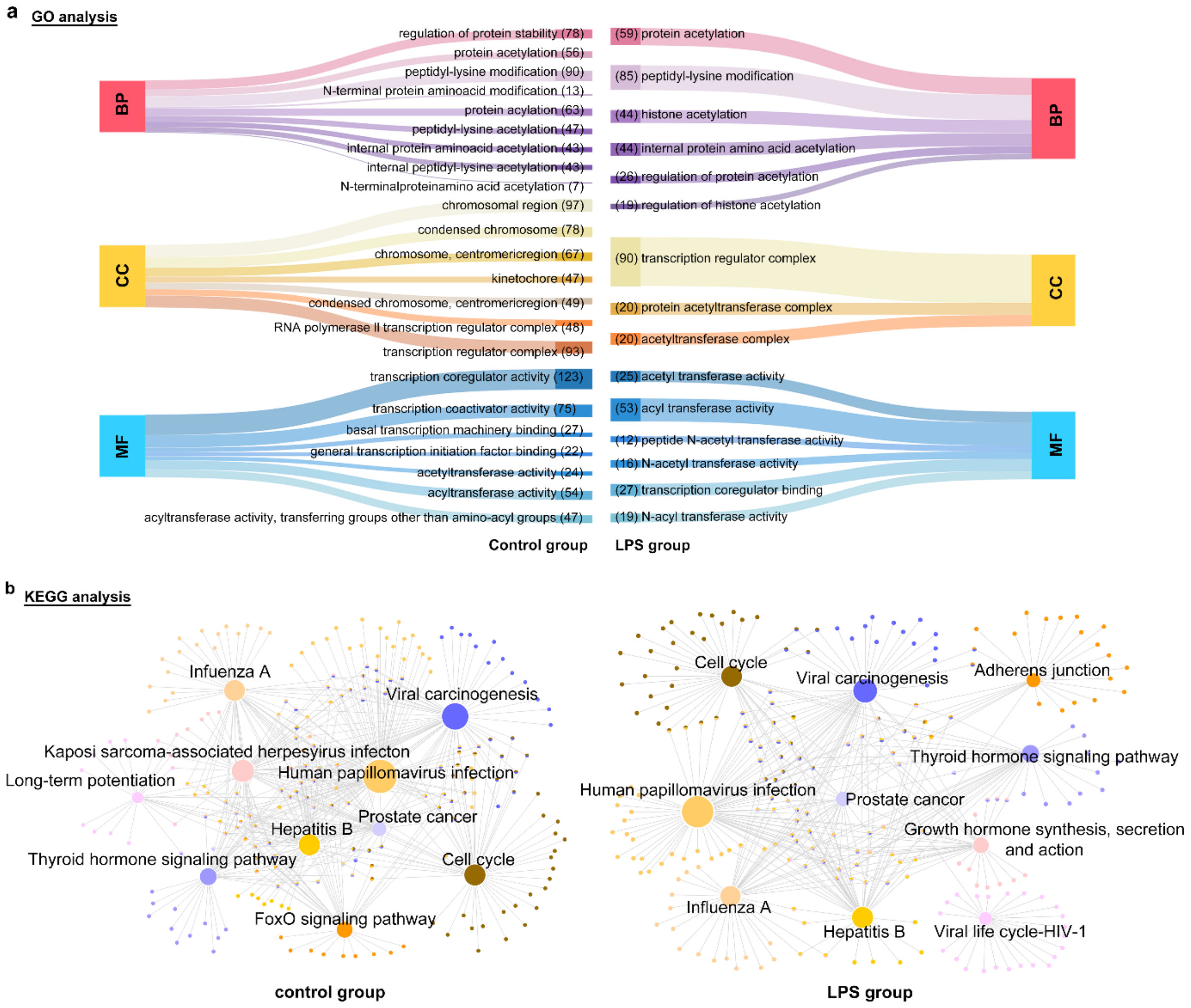

Supplementary Fig. 6 Enrichment analysis of proteins potentially interacting with CPT1B

(a) GO enrichment results for the potential interaction proteins with CPT1B in control and LPS-treated groups, showing biological process (BP), molecular function (MF), and cellular component (CC) terms. **(b)** KEGG pathway enrichment analysis for the potential interaction proteins with CPT1B in control and LPS-treated groups.

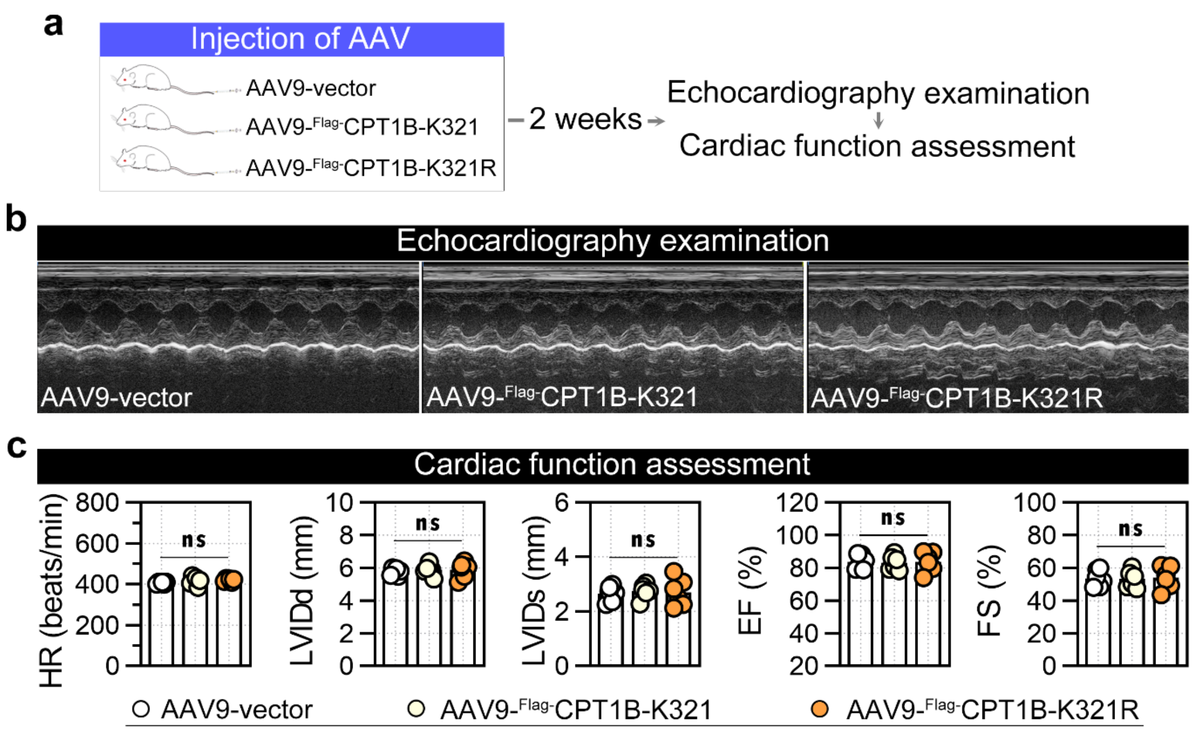

Supplementary Fig. 7 Effects of CPT1B K321cr on healthy rats

(a) Experimental design for cardiac-specific AAV9 delivery in rats. **(b)** Representative M-mode echocardiograms. **(c)** Echocardiographic assessment of the heart rate (HR), left ventricular internal diameter in diastole (LVIDd) and systole (LVIDs), ejection fraction (EF), and fractional shortening (FS). Data are presented as mean ± SD. ns, not significance.
