# Supplementary material for: CPT1B K321 crotonylation contributes to cardiac dysfunction in endotoxic shock
Source: Exp Mol Med. 2026 May 28;58(5):1674–87. doi: 10.1038/s12276-026-01730-2 (PMC13233815; doi:10.1038/s12276-026-01730-2)
Supplement: Supplementary file 3 — Source Data [file 12276_2026_1730_MOESM3_ESM.pdf]

Figure 3H

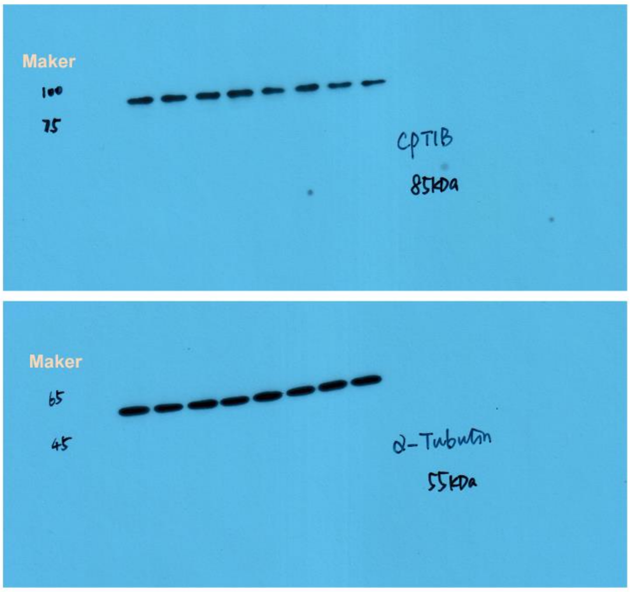

Figure 3I

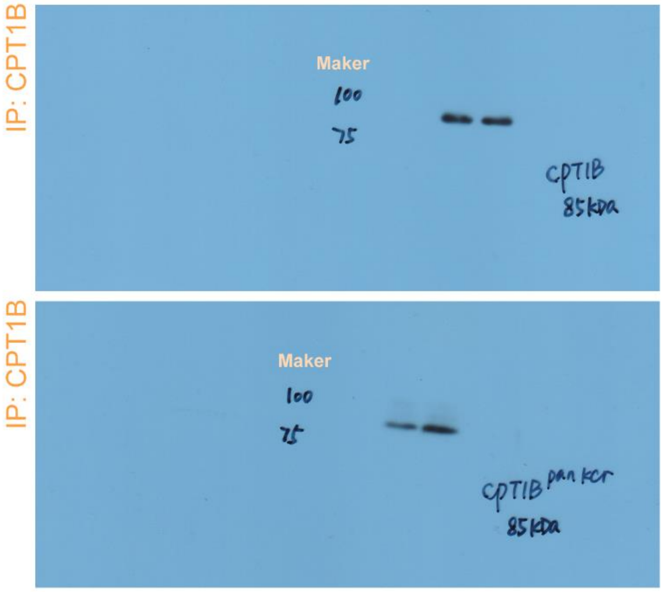

Figure 3J

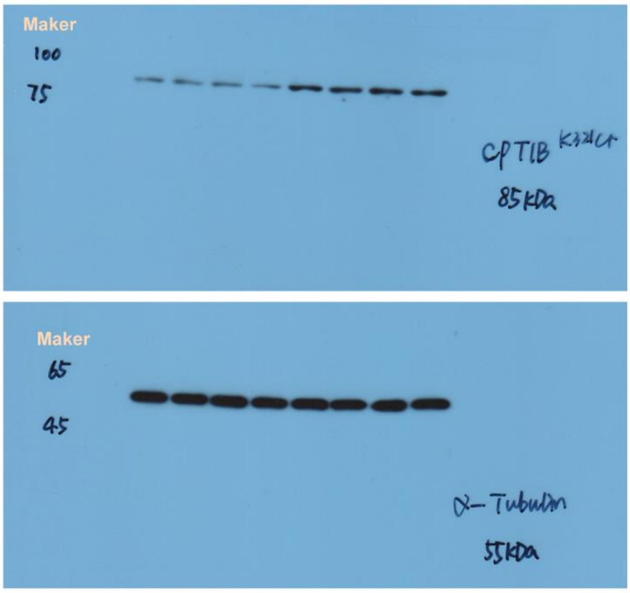

Figure 4B

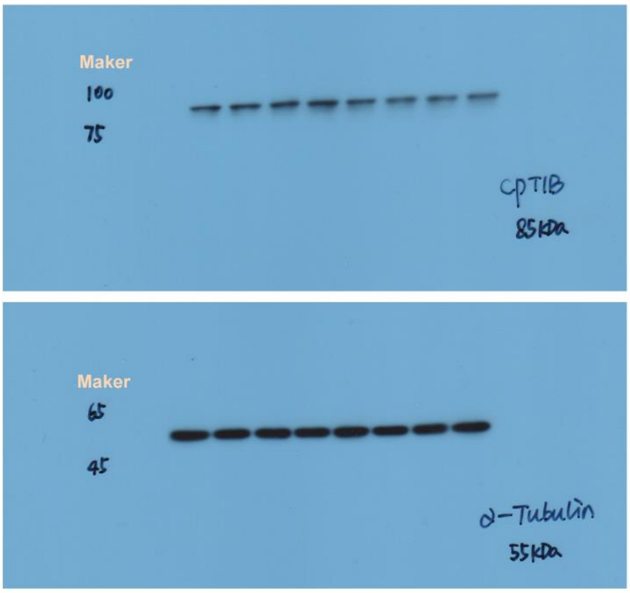

Figure 4C

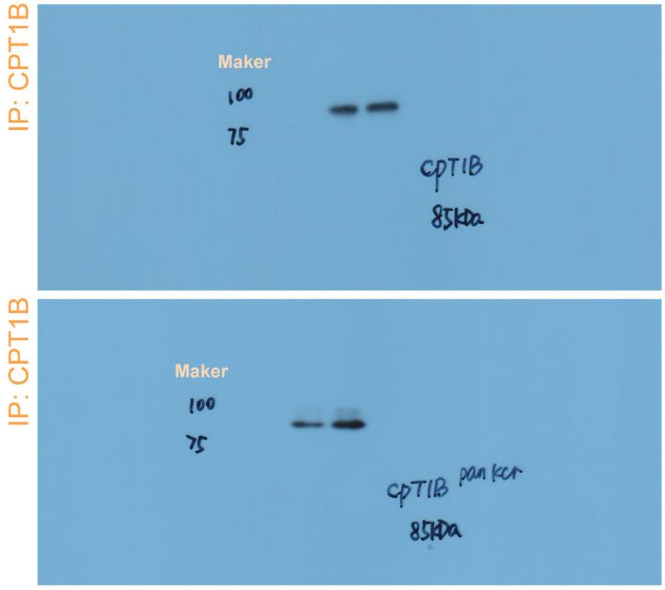

Figure 4D

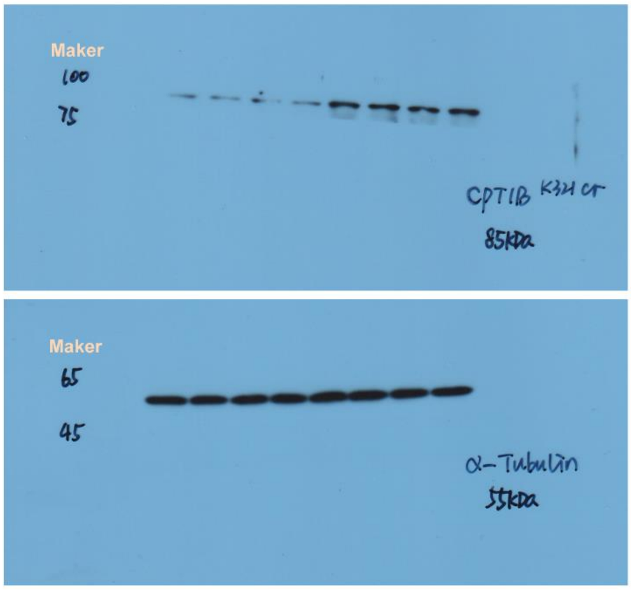

Figure 5A

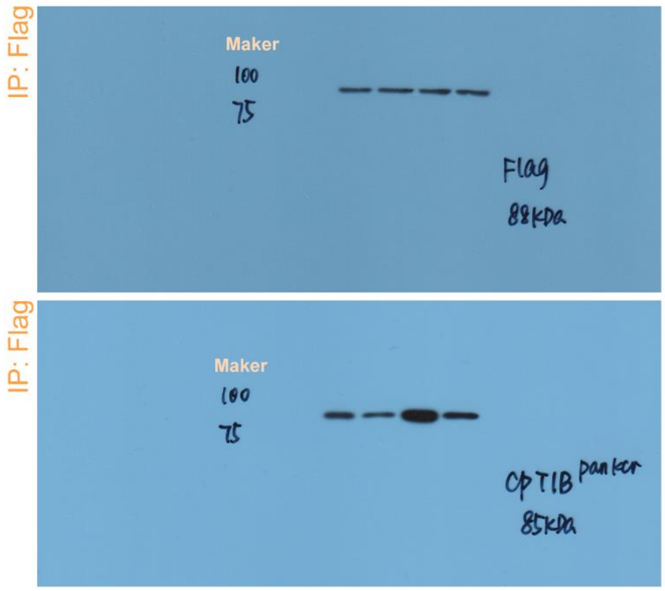
IP: Flag
Maker
100
75
Flag
88kDa
IP: Flag
Maker
100
75
CPT1B
85kDa

Figure 6C

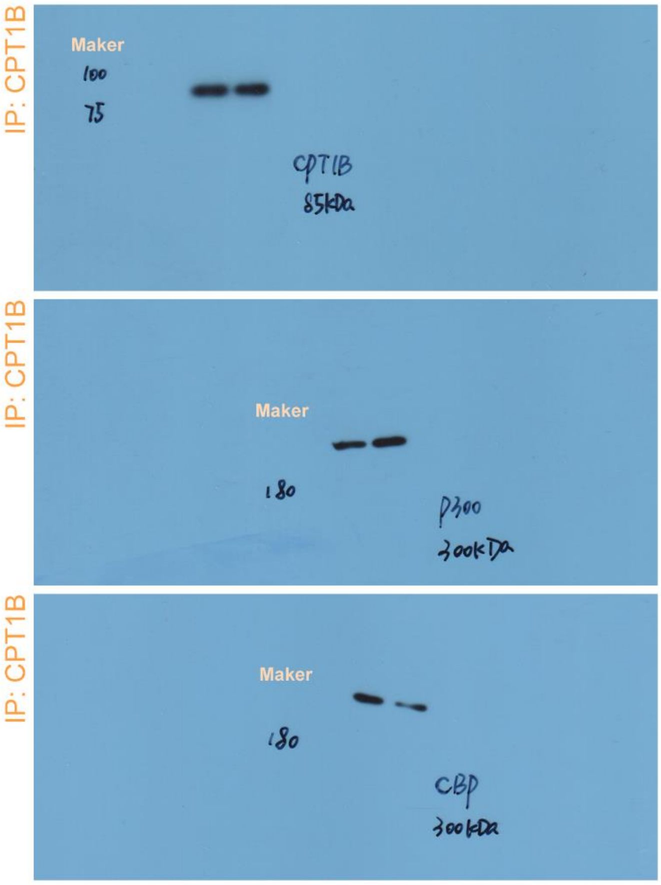
IP: CPT1B
Maker
100
75
CPT1B
85kDa
IP: CPT1B
Maker
180
p300
300kDa
IP: CPT1B
Maker
180
CBP
300kDa

Figure 6F

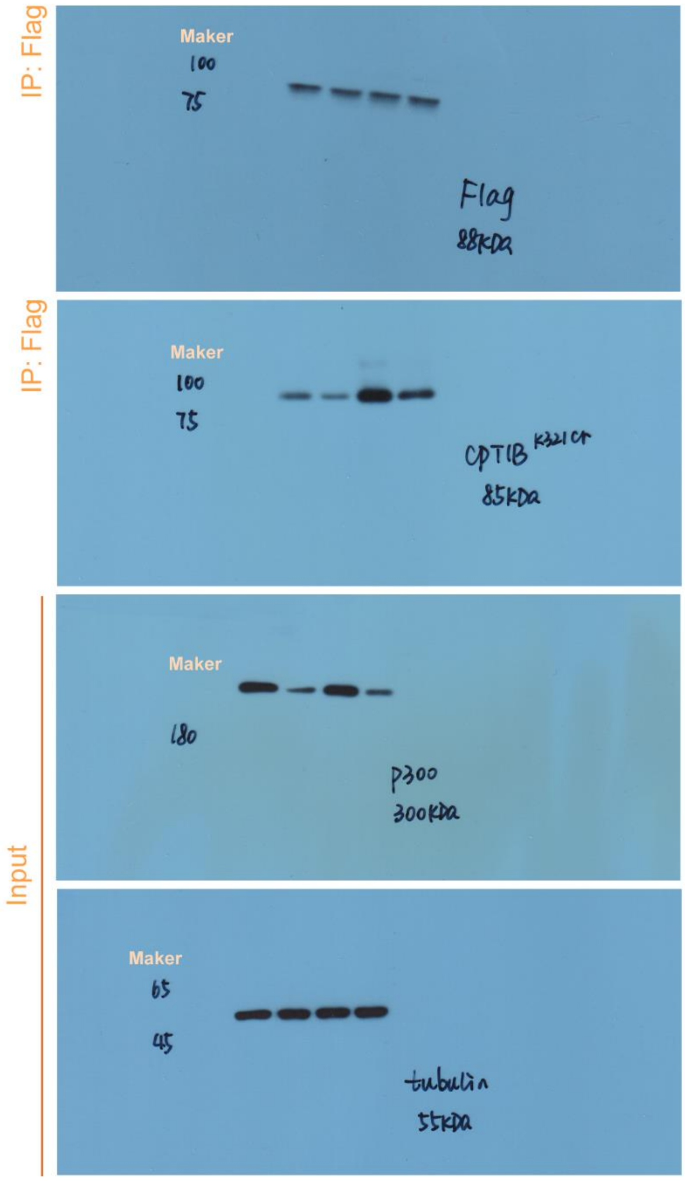
IP: Flag
Maker
100
75
Flag
88KDa
IP: Flag
Maker
100
75
CPT1B K321cr
85KDa
Input
Maker
180
P300
300KDa
Maker
65
45
tubulin
55KDa

Figure 6G

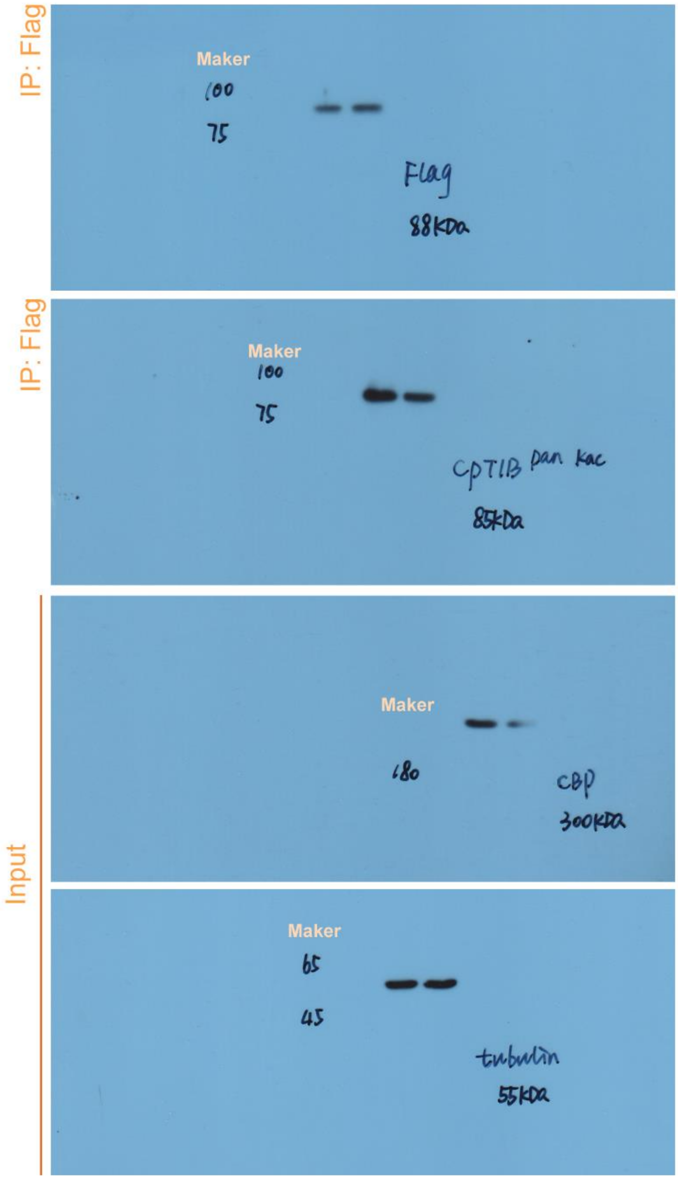
IP: Flag
Maker
100
75
Flag
88KDa
IP: Flag
Maker
100
75
CPT1B pan Kac
85kDa
Input
Maker
180
CBP
300KDa
Maker
65
45
tubulin
55KDa

Figure 6H

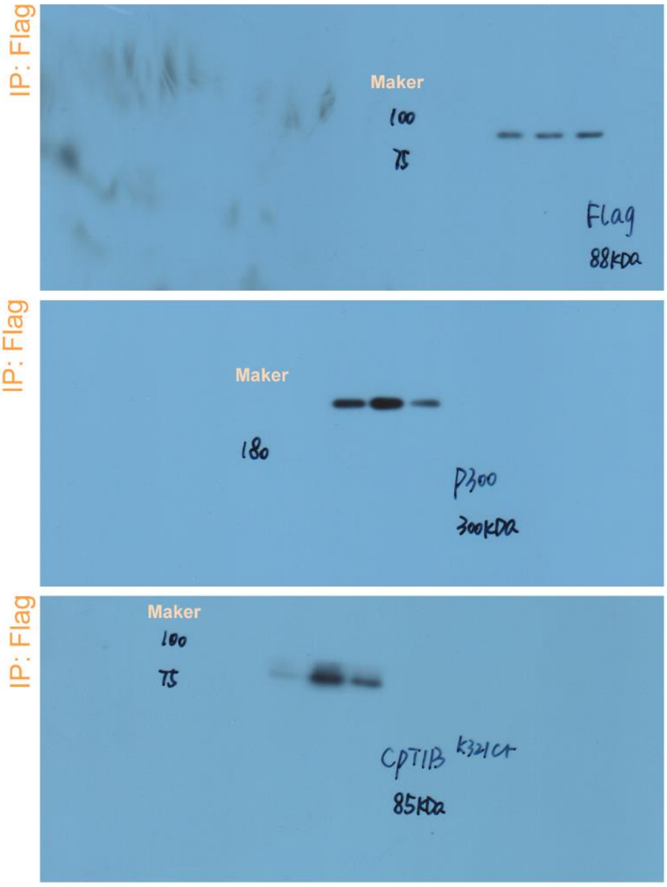

IP: Flag
Maker
100
75
Flag
88KDa
IP: Flag
Maker
180
P300
300KDa
IP: Flag
Maker
100
75
CPT1B K321Cr
85KDa

Figure S3B

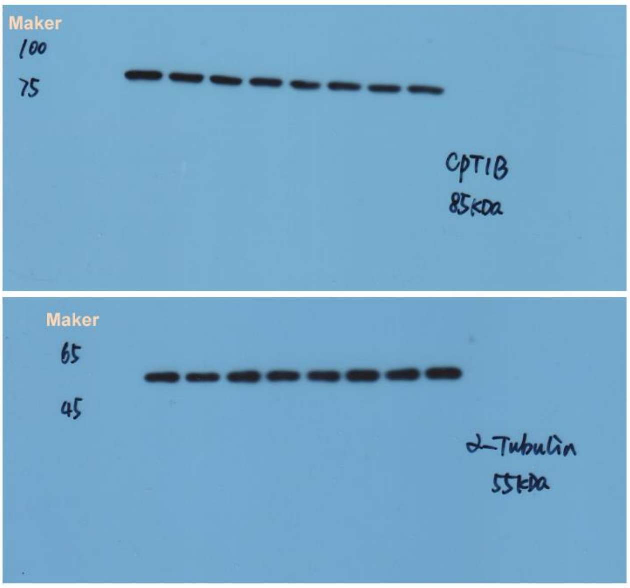

Figure S3C

IP: CPT1B

IP: CPT1B

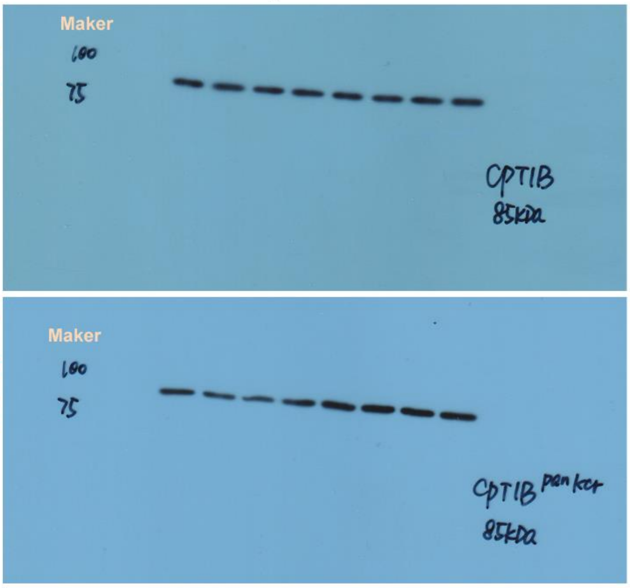

Figure S3D

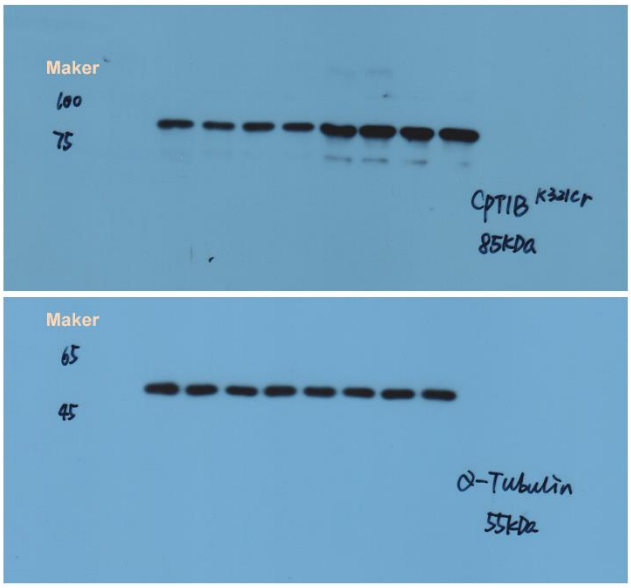

Figure S4A

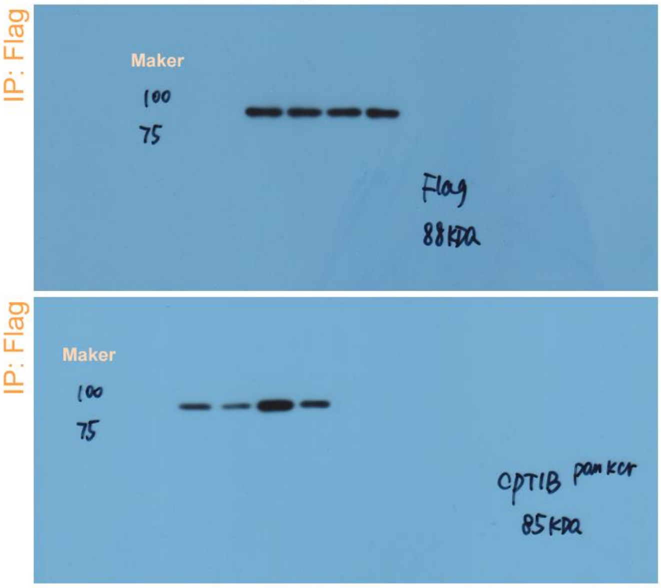

Figure S5A

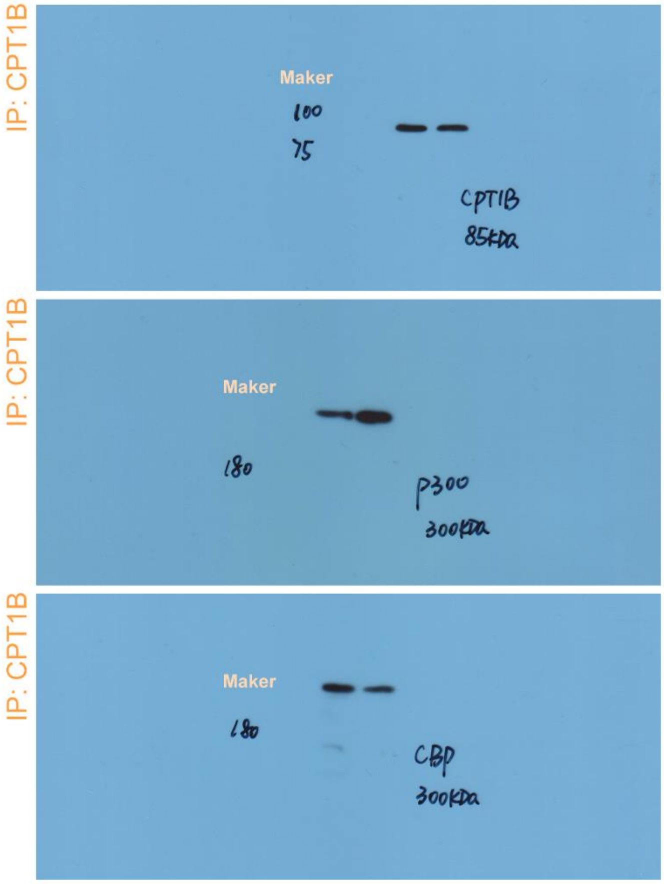
IP: CPT1B
Maker
100
75
CPT1B
85KDa
IP: CPT1B
Maker
180
P300
300KDa
IP: CPT1B
Maker
180
CBP
300KDa

Figure S5B

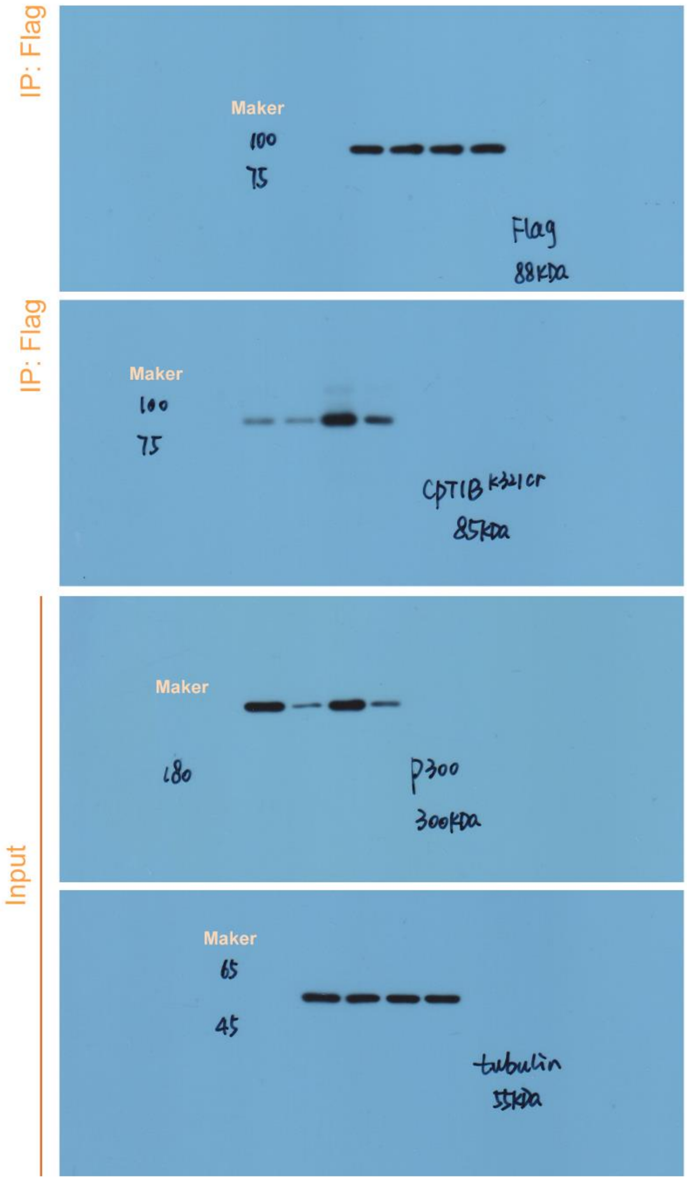

IP: Flag
Maker
100
75
Flag
88KDa
IP: Flag
Maker
100
75
CPT1B K321cr
85KDa
Input
Maker
180
P300
300KDa
Maker
65
45
tubulin
55KDa

Figure S5C

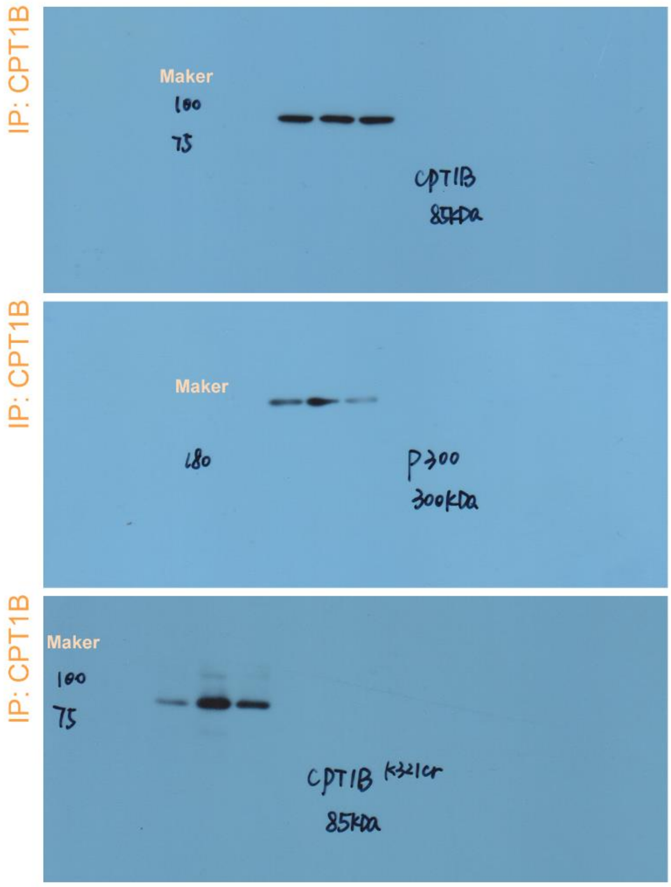
IP: CPT1B
Maker
100
75
CPT1B
85KDa
IP: CPT1B
Maker
180
P300
300KDa
IP: CPT1B
Maker
100
75
CPT1B K321cr
85KDa
